# Analysis of the Population Structure of *Anaplasma phagocytophilum* Using Multilocus Sequence Typing

**DOI:** 10.1371/journal.pone.0093725

**Published:** 2014-04-03

**Authors:** Christian Huhn, Christina Winter, Timo Wolfsperger, Nicole Wüppenhorst, Katja Strašek Smrdel, Jasmin Skuballa, Miriam Pfäffle, Trevor Petney, Cornelia Silaghi, Viktor Dyachenko, Nikola Pantchev, Reinhard K. Straubinger, Daniel Schaarschmidt-Kiener, Martin Ganter, Matthew L. Aardema, Friederike D. von Loewenich

**Affiliations:** 1 Institute of Medical Microbiology and Hygiene, University of Freiburg, Freiburg, Germany; 2 Institute of Microbiology and Immunology, Faculty of Medicine, University of Ljubljana, Ljubljana, Slovenia; 3 Department of Ecology and Parasitology, Karlsruhe Institute of Technology, Karlsruhe, Germany; 4 Comparative Tropical Medicine and Parasitology, Ludwig-Maximilians-University Munich, Munich, Germany; 5 Institute for Infectious Diseases and Zoonoses, Ludwig-Maximilians-University Munich, Munich, Germany; 6 IDEXX Vet Med Lab, Ludwigsburg, Germany; 7 Laboratory at Zugersee, Hünenberg, Switzerland; 8 Clinic for Swine and Small Ruminants, University of Veterinary Medicine Hannover, Hannover, Germany; 9 Department of Ecology and Evolutionary Biology, Princeton University, Princeton, New Jersey, United States of America; The Johns Hopkins University School of Medicine, United States of America

## Abstract

*Anaplasma phagocytophilum* is a Gram-negative obligate intracellular bacterium that replicates in neutrophils. It is transmitted via tick-bite and causes febrile disease in humans and animals. Human granulocytic anaplasmosis is regarded as an emerging infectious disease in North America, Europe and Asia. However, although increasingly detected, it is still rare in Europe. Clinically apparent *A. phagocytophilum* infections in animals are mainly found in horses, dogs, cats, sheep and cattle. Evidence from cross-infection experiments that *A. phagocytophilum* isolates of distinct host origin are not uniformly infectious for heterologous hosts has led to several approaches of molecular strain characterization. Unfortunately, the results of these studies are not always easily comparable, because different gene regions and fragment lengths were investigated. Multilocus sequence typing is a widely accepted method for molecular characterization of bacteria. We here provide for the first time a universal typing method that is easily transferable between different laboratories. We validated our approach on an unprecedented large data set of almost 400 *A. phagocytophilum* strains from humans and animals mostly from Europe. The typability was 74% (284/383). One major clonal complex containing 177 strains was detected. However, 54% (49/90) of the sequence types were not part of a clonal complex indicating that the population structure of *A. phagocytophilum* is probably semiclonal. All strains from humans, dogs and horses from Europe belonged to the same clonal complex. As canine and equine granulocytic anaplasmosis occurs frequently in Europe, human granulocytic anaplasmosis is likely to be underdiagnosed in Europe. Further, wild boars and hedgehogs may serve as reservoir hosts of the disease in humans and domestic animals in Europe, because their strains belonged to the same clonal complex. In contrast, as they were only distantly related, roe deer, voles and shrews are unlikely to harbor *A. phagocytophilum* strains infectious for humans, domestic or farm animals.

## Introduction

*Anaplasma phagocytophilum* is a Gram-negative obligate intracellular bacterium that replicates in neutrophils [1]. It is transmitted via tick-bite and causes febrile disease in humans and animals. The main vectors of *A. phagocytophilum* are *Ixodes ricinus* in much of Europe, *I. scapularis* and *I. pacificus* in North America and *I. persulcatus* in Eastern Europe and East Asia [2].

Human granulocytic anaplasmosis (HGA) was first recognized in the USA in 1994 [3] and has reached its largest reported incidence since it became a notifiable disease in this country with 2,575 cases in 2011 [4]. Although increasingly detected, it is still a rare disease in Europe [5], [6]. Underreporting, differences in host range or a reduced virulence in humans of the *A. phagocytophilum* strains circulating in Europe might account for this phenomenon. HGA also occurs in China [7], [8]. However, the person to person transmission [9] and a mortality rate of 26.5% [10] initially described in Chinese patients, were later on found to be caused by a novel bunyavirus [7], [11], [12].

Clinically apparent *A. phagocytophilum* infections in animals are mainly found in domestic animals such as horses [13], dogs [14] and cats [15] as well as in farm animals such as sheep, cattle and goats [16]. Since *A. phagocytophilum* is not transmitted transovarially, at least in *Ixodes* spp. ticks [17], it is generally thought to depend on reservoir hosts to complete its life cycle. Whereas the white-footed mouse (*Peromyscus leucopus*) is a well characterized reservoir species in North America [18], [19], the situation is less clear in Europe. Although *A. phagocytophilum* has been detected in a wide range of wildlife animals in Europe including wild ruminants, wild boars, red foxes and small mammals, their respective reservoir competence has not been fully established [20].

Evidence from cross-infection experiments indicating that *A. phagocytophilum* isolates of distinct host origin are not uniformly infectious for heterologous hosts has led to several approaches for molecular strain characterization [21], [22]. The target genes that have been used most often comprise the 16S rRNA, *groESL*, *ankA*, *msp2* and *msp4* genes [22]. Unfortunately, the results of the studies performed to date are not always easily comparable because different gene regions and fragment lengths have been investigated. In contrast, multilocus sequence typing (MLST) offers the advantage of a standardized approach, a universal nomenclature and a free data exchange via the internet [23]. Therefore, we chose to develop a MLST scheme for *A. phagocytophilum*.

A total of 380 *A. phagocytophilum* positive samples from humans, animals and *I. ricinus* ticks from Europe were included in our data set. 11 additional samples originated from the USA. The MLST data we generated were used to analyze the population structure of *A. phagocytophilum*, to assess the concordance between MLST, 16S rRNA and *ankA* gene-based typing, to test whether particular *A. phagocytophilum* strains are associated with distinct host species and to examine which wildlife species may be potential reservoir hosts for granulocytic anaplasmosis in humans, domestic and farm animals in Europe.

## Materials and Methods

### Ethics Statement

The samples from humans, domestic and farm animals were obtained as part of routine diagnostic evaluations [24], [25], [26], [27]. All samples were de-identified. Written informed consent was obtained form the patients and owners, respectively. A part of the ovine samples was collected for the Q-fever surveillance program of the federal state of Lower-Saxony [24]. The red foxes were killed for the official rabies monitoring program of the federal state of Brandenburg [28]. All other wild large animals were shot by professional hunters during the annual hunting seasons [24]. Hedgehogs from a captive population in the federal state of Baden-Württemberg and from hedgehog caretaking stations in various areas of Germany were bled from the saphenous vein or died naturally [29], [30]. None of the hedgehogs was sacrificed for this study. For Germany, permission to trap rodents using snap traps was given by the District Government Stuttgart, Germany [31]. For the United Kingdom, protocols for the handling and sampling of wild small mammals were approved by the University of Liverpool Committee on Research Ethics and were conducted in compliance with the terms and conditions of licenses awarded under the UK Government Animals (Scientific Procedures) Act, 1986 [32], [33].

### Samples

A total of 391 *A. phagocytophilum* positive DNA samples from 42 humans, 93 domestic animals (63 dogs, 28 horses, 2 cats), 62 farm animals (54 sheep, 7 cattle, 1 goat), 99 large wild animals (49 roe deer, 18 red deer, 15 European bison, 12 wild boars, 3 chamois, 2 red foxes), 61 small mammals (34 European hedgehogs, 24 voles, 3 shrews) and 34 *I. ricinus* ticks were included. The 34 hedgehog samples originated from 24 individuals, because 6 of them were sampled several times. 380 samples were derived from different European countries and 11 from the USA. The geographic origin of the samples is shown in Table S1.

### MLST Analysis

For our MLST analysis seven housekeeping genes (*pheS, glyA, fumC, mdh, sucA, dnaN, atpA*) each located at least 10 kb apart in the *A. phagocytophilum* HZ genome (GenBank accession number CP000235) were chosen. Sequencing revealed that all of them contained more synonymous than non-synonymous mutations. Fragments of an in-frame length of approximately 400 bp (without primer sequences) were amplified by nested PCR. 2 μl of DNA were used as a template in a 50-μl reaction mixture containing 50 mM KCl, 20 mM Tris-HCl (pH 8.4), 2 mM MgCl_2_, 0.2 mM deoxynucleoside triphosphates, 0.4 μM concentrations of each primer and 0.2 μl (1 U) of *Taq* DNA polymerase (Invitrogen, Karlsruhe, Germany). PCRs were performed by using a GeneAmp PCR System 9700 (Applied Biosystems, Darmstadt, Germany) under the following conditions: an initial denaturation at 94°C for 3 min, followed by 40 cycles of denaturation at 94°C for 30 s, annealing at 54°C for 30 s, extension at 72°C for 30 s and a final extension at 72°C for 10 min. Because of nucleotide polymorphisms a set of multiple primers had to be developed for amplification (Table S2). The nucleotide sequences of the primers are shown in Table S3. Nested PCR products were directly sequenced bidirectionally using a 3130 Genetic Analyzer (Applied Biosystems) and a BigDye Terminator v3.1 cycle sequencing kit (Applied Biosystems). Forward and reverse sequences were aligned and quality controlled using TraceEdit Pro 1.2.1 (Ridom, Würzburg, Germany). Different sequences of a given locus were ascribed a unique, but arbitrary allele number and each unique combination of alleles was assigned a sequence type (ST). In 383 samples, all seven housekeeping genes could be amplified. However, in 3 roe deer, 2 sheep, 1 dog, 1 red deer and 1 hedgehog samples the DNA was depleted before completion. The *pheS, glyA, fumC, mdh, sucA, dnaN* and *atpA* sequences were assigned GenBank accession numbers KF242733 to KF245413. The *A. phagocytophilum* MLST profiles and isolate databases are available at http://pubmlst.org/aphagocytophilum/.

### 16S rRNA and *ankA* Gene Sequences

Partial 16S rRNA and complete *ankA* gene sequences of 256 samples were determined as part of previous studies [24], [25], [27], [28], [29], [30]. In 135 additional samples, the *A. phagocytophilum* 16S rRNA gene was amplified and sequenced as described earlier [34], . Only in 130 of these, nested PCR amplification and sequencing of partial *ankA* sequences was possible, because the DNA was used up in 4 hedgehog and 1 tick samples. The composition of the PCR reaction mixture was the same as described above. The following PCR conditions were used: an initial denaturation at 94°C for 3 min, followed by 40 cycles of denaturation at 94°C for 30 s, annealing at 52°C for 30 s, extension at 72°C for 1 min (first amplification) and for 30 s (nested amplification), respectively and a final extension at 72°C for 10 min. Primers SLO fo 1 (5′-GGG ATR AGT GCR GTG CAG YAT-3′) and SLO re 1 (5′-ACT GCR GCM GCT ARA GGR CT-3′) were used in the first amplification step and primers SLO fo 2 (5′-TTA CGC TGT RRT RGC ATR GAC-3′) and SLO re 2 (5′-AWR GWT CCS KYA GGA GYA TTT A-3′) in the nested amplification step. Nested PCR products were directly sequenced bidirectionally as described above. Sequences were edited and assembled with the SeqMan program 8.02 of the DNASTAR package (Lasergene, Madison, WI). The 16S rRNA and *ankA* gene sequences are available at GenBank under the accession numbers KF242536 to KF242732, KF286395 and JQ347533 to JQ347591.

### Allele-based Analyses

The allele-based analyses were carried out by using eBURST (http://eburst.mlst.net/) and START2 (http://pubmlst.org/software/analysis/start2/). Clonal complexes were defined by sharing identical alleles at five of the seven loci with at least one other member of the group. The standardized index of association [36] was calculated in order to detect linkage disequilibrium.

### Sequence-based Analyses

For phylogenetic analyses the program MEGA 5.1 [37] was used. Sequences were aligned by ClustalW applying the IUB matrix (16S rRNA gene) or codon-aligned applying the PAM (Dayhoff) matrix (concatenated allele sequences, *ankA*). Tree construction was achieved by the maximum-likelihood (ML) method using the Tamura-Nei model and a gamma distribution with the use all sites option. Bootstrap analysis was conducted with 1,000 replicates. Net average distances between concatenated allele gene clusters and *ankA* gene clusters were computed using the Jukes-Cantor matrix and applying the complete deletion option.

### Structure Analysis

The program STRUCTURE 2.3.4 [38] was used to examine population clustering among the *A. phagocytophilum* strains investigated. A dataset of 518 variable sites was created consisting of all single-nucleotide polymorphisms found in the seven housekeeping genes (variable indels were not included in this dataset). With these single-nucleotide polymorphisms, we examined the number of clusters (K) represented in the data from 1 to 9, using the admixture model. We applied a burn-in period of 10,000 followed by 20,000 replications. Each value of K (1–9) was run five independent times. The program STRUCTURE HARVESTER 0.6.93 [39] was used to analyze these results and to estimate what number of clusters is best supported by the data applying Evanno’s ΔK [40]. The program DISTRUCT 1.1 [41] was used to visualize the clusters.

### Analyses for Recombination

Four separate analyses were used to test for recombination in each of the seven housekeeping genes and *ankA*. These were GENECONV 1.81 (available at http://www.math.wustl.edu/~sawyer/geneconv/index.html) [42], the pairwise homoplasy index (PHI) [43], maximum χ^2^ [44] and the neighborhood similarity score (NSS) [45]. These last three methods were applied using the PhiPack package [43]. Using the eight datasets of sequences aligned by ClustalW as described above, regions with indel polymorphisms and sequences containing ambiguous sites were removed first. Additionally, when two or more sequences were identical, only a single version was retained. In the GENECONV analyses, recombination was assessed using only silent site polymorphisms. The number of mismatches allowed between recombining fragments (gscale) was set to 0 and putative recombinant regions were determined for inner fragments using 10,000 random permutations. A window size of 50 nucleotides was used in the PHI analyses. In the maximum χ^2^ tests, a fixed window size of 2/3 the number of polymorphic sites was applied. *p* values were estimated based on 1,000 permutations for PHI, maximum χ^2^ and NSS with significance at α <0.05.

### Analyses for Selection

To examine selection, the non-synonymous (dN) and synonymous (dS) rate ratios (dN/dS) [46] for each of the seven housekeeping genes and *ankA* were assessed using START2. For *ankA* we also looked for evidence of site-specific selection in this gene. To do so, the CODEML program in PAML 4 [47] was applied. Using the tree topology generated from our MLST analysis, two site-specific models that allow dN/dS rate ratios (ω = dN/dS) to vary among codons were applied. These models were M1a (nearly neutral), which only allows site classes for ω to vary between 0 and 1 and M2a (positive selection), which allows an additional site class for ω >1 [48]. A likelihood ratio test (LRT) comparing these two models to a χ^2^ distribution (degrees of freedom = 2) was carried out to determine whether there is significant evidence for positive selection in the *ankA* gene. The Bayes empirical Bayes method (BEB) as implemented in CODEML was used to identify sites that have evolved under positive selection [49].

### Comparison of Typing Methods

Typability, discriminatory indices and Wallace coefficients [50] were calculated using Ridom EpiCompare (http://www.ridom.de/epicompare/) and adjusted Wallace coefficients [51] using the online tool available at: http://darwin.phyloviz.net/ComparingPartitions/index.php?link=Tool.

## Results

### Allele-based MLST Analysis

In 8 samples the DNA was depleted before completion. In 383 of the 391 samples, all seven housekeeping genes could be amplified. However, the analysis was hindered by the fact that numerous chromatograms contained double peaks, a phenomenon that was most prominent in samples from roe deer, red deer and European bison. Double peaks were almost exclusively restricted to variable sites and were generally present in more than one of the seven alleles of a given sample. A total of 99 samples showing ambiguous nucleotides had to be excluded from the allele-based MLST analysis. Thus, only in 284 cases a sequence type (ST) could be ascribed. In total, 90 different STs were detected. 67 (74%) of them were found only once and thus were unique. The distribution of STs among the different host species is shown in Table 1. The allele frequency and the number of polymorphic sites for each locus are shown in Table 2.

**Table 1:** ST and host species of the *A. phagocytophilum* positive samples (n = 383).

ST	Number^1^	Host species	ST	Number	Host species
NT^2^	99	34 (46) roe deer^3^	79	3	3 (44) sheep
		14 (17) red deer	115	3	3 (20) ticks
		9 (15) European bison	3	2	2 (44) sheep
		9 (33) hedgehogs	15	2	2 (6) European bison
		8 (52) sheep	27	2	2 (12) roe deer
		7 (24) voles	50	2	2 (44) sheep
		2 (3) chamois	57	2	1 (61) dogs
		1 (62) dogs			1 (28) horses
		1 (7) cattle	65	2	2 (44) sheep
		14 (34) ticks	75	2	2 (44) sheep
25	73	30 (42) humans^4^	80	2	2 (44) sheep
		26 (61) dogs	104	2	1 (12) roe deer
		9 (12) wild boars			1 (34) ticks
		7 (28) horses	140	2	2 (6) cattle
		1 (2) cats	142	2	2 (17) voles
55	47	21 (24) hedgehogs	145	2	2 (17) voles
		16 (61) dogs	149	2	2 (17) voles
		1 (42) humans	153	2	2 (3) shrews
		1 (2) cats	unique	67	24 (44) sheep
		1 (2) red foxes			9 (12) roe deer
		7 (20) ticks			5 (61) dogs
54	34	20 (28) horses			4 (6) cattle
		10 (61) dogs			4 (6) European bison
		3 (44) sheep			4 (17) voles
		1 (12) wild boars			3 (3) red deer
64	10	9 (42) humans			2 (42) humans
		1 (61) dogs			2 (12) wild boars
58	8	3 (24) hedgehogs			1 (1) goat
		2 (61) dogs			1 (1) chamois
		3 (20) ticks			1 (2) red foxes
143	7	7 (17) voles			1 (3) shrews
7	4	4 (44) sheep			6 (20) ticks

1 Prevalence of the respective STs in the dataset.

2 NT = nontypeable.

3 n (n) = nontypeable strains (all strains).

4 n (n) = strains with respective ST (typeable strains).

**Table 2:** Allele frequency and number of polymorphic sites of the seven loci of the *A. phagocytophilum* MLST scheme^1^ and of the partial 16S rRNA^2^ and *ankA* 3 gene sequences.

Gene	In-frame fragment length (bp)	Allele frequency	Polymorphic sites (%)	dN/dS
*pheS*	438	44	86 (20)	0.0505
*glyA*	387	33	70 (18)	0.0512
*fumC*	411	32	56 (14)	0.0418
*mdh*	387	22	44 (11)	0.0706
*sucA*	429	36	83 (19)	0.1127
*dnaN*	405	36	69 (17)	0.0419
*atpA*	420	27	78 (19)	0.0439
16S rRNA gene	497	14	9 (2)	na^4^
*ankA*	519–537	93	330 (61)	0.5884

1 284 strains typeable by MLST were included.

2 369 strains without ambiguous nucleotides were included.

3 369 sequences without ambiguous nucleotides from 363 samples were included.

4 not applicable.

STs can be grouped into clonal complexes by similarity of their allelic profile [23]. In our data set, we defined a clonal complex by sharing identical alleles at five of the seven loci with at least one other member of the group. The relationship between the STs was analyzed using eBURST (http://eburst.mlst.net/), which identified eight clonal complexes (Table 3). 223 of 284 (79%) *A. phagocytophilum* strains belonged to a clonal complex. However, with the exception of clonal complexes 1, 2 and 5, the other clonal complexes contained less than 10 strains. The most prominent clonal complex 1 contained 177 strains. ST 25 was identified as a founding genotype, because it differed from the largest number of other STs of clonal complex 1 at only one single locus. This was supported by a bootstrap value of 67% (1,000 replicates) and suggests it may resemble the common ancestor of the other STs. All typeable strains from humans and dogs from Europe and all typeable strains from horses, hedgehogs, wild boars, cats and red foxes belonged to clonal complex 1 (Table 4). The same was true for a proportion of the tick strains and for a minority of sheep strains. In contrast, all 11 strains from the USA (10 human, 1 canine) were part of clonal complex 5. Interestingly, *A. phagocytophilum* strains from clonal complexes 1 and 5 did not share any of the alleles, indicating a longer evolutionary distance between strains from Europe and North America. All typeable strains from voles and shrews belonged exclusively to clonal complex 2. Thus, the allele-based MLST analysis proved to be informative with respect to host origin and geographic provenance.

**Table 3:** Clonal complexes, respective STs and their prevalence in the data set.

Clonal complex^1^	ST^2^	Number^3^	Clonal complex	ST	Number
1 (n = 16)^4^	25	73	3 (n = 4)	11	1
	55	47		13	1
	54	34		98	1
	58	8		100	1
	57	2	4 (n = 3)	15	2
	115	3		140	2
	66	1		173	1
	67	1	5 (n = 2)	64	10
	68	1		161	1
	116	1	6 (n = 2)	1	1
	120	1		136	1
	134	1	7 (n = 2)	70	1
	162	1		71	1
	163	1	8 (n = 2)	61	1
	164	1		62	1
	188	1			
2 (n = 10)	143	7			
	142	2			
	145	2			
	149	2			
	153	2			
	141	1			
	144	1			
	147	1			
	152	1			
	155	1			

1 Clonal complexes were defined by sharing identical alleles at five of the seven loci with at least one other member of the group.

2 41 of the 90 STs belonged to a clonal complex, whereas 49 STs were found to be unlinked.

3 Prevalence of the respective STs in the data set.

4 The number of different STs forming a clonal complex is given in parenthesis.

**Table 4:** Clonal complexes and host species of the *A. phagocytophilum* strains typeable by MLST (n = 284).

Clonal complex^1^	Number^2^	Host species^3^	Clonal complex	Number	Host species
1	177	60^4^ (61) dogs	5	11	10^6^ (42) humans
		32^5^ (42) humans			1^7^ (61) dogs
		28 (28) horses	6	2	2 (44) sheep
		24 (24) hedgehogs	7	2	2 (44) sheep
		12 (12) wild boars	8	2	2 (12) roe deer
		3 (44) sheep	without	61	37 (44) sheep
		2 (2) cats			10 (12) roe deer
		2 (2) red foxes			3 (3) red deer
		14 (20) ticks			2 (6) cattle
2	20	17 (17) voles			2 (6) European bison
		3 (3) shrews			1 (1) chamois
3	4	2 (6) cattle			6 (20) ticks
		2 (6) European bison			
4	5	2 (6) cattle			
		2 (6) European bison			
		1 (1) goat			

1 Clonal complexes were defined by sharing identical alleles at five of the seven loci with at least one other member of the group.

2 Prevalence of strains in the data set.

3 n (n) = strains belonging to the respective clonal complex (typeable strains).

4 60 (60) dogs from Europe.

5 32 (32) humans from Europe.

6 10 (10) humans from the USA.

7 1 (1) dog from the USA.

In a fully clonal population that reproduces asexually without recombination taking place, mutations will accumulate in nonrandom combinations. This situation is called linkage disequilibrium meaning that two or more alleles are observed together in a genome more frequently than by chance alone [52]. Calculating the standardized index of association [36] significant linkage disequilibrium was detected in our data set indicating a clonal population structure.

However, only 41 (46%) of the 90 STs defined so far were part of a clonal complex, whereas 49 (54%) were found to be unlinked. In contrast to European strains from dogs, humans and horses, which uniformly belonged to clonal complex 1, strains from sheep and roe deer showed a marked heterogeneity with a high proportion of unlinked STs (Figure S1). The population snapshot shown in Figure S1 thus indicates that the population structure of *A. phagocytophilum* might be semiclonal with a high proportion of relatively rare unlinked STs superimposed by a limited number of rather frequent genotypes.

### Sequence-based MLST Analysis

Allele and ST numbers themselves are uninformative concerning genetic relationship, because an allele change does not reflect the number of nucleotide exchanges. Thus, the eBURST analysis is rather independent of the sequences as it takes into account only the allelic profiles. Furthermore, in 99 (26%) of the 383 samples the allele-based analysis could not be applied because they contained ambiguous nucleotides. We therefore constructed a maximum-likelihood (ML) phylogenetic tree using the use all sites option.

This sequence-based phylogenetic analysis revealed that *A. phagocytophilum* strains from humans, dogs, horses, wild boars and hedgehogs were very uniform, whereas strains from ruminants such as sheep, cattle, European bison, roe deer and red deer showed a much higher degree of heterogeneity (Figure S2 A). Three major clusters could be distinguished. Cluster 2 contained all 46 roe deer strains and 1 of the 17 red deer strains. All 24 vole and all 3 shrew strains were part of cluster 3, which is equivalent to clonal complex 2. All other *A. phagocytophilum* strains from humans, hedgehogs, domestic, farm and large wild animals belonged to cluster 1. Strains from the USA were found in cluster 1 but on a separate branch supported by a bootstrap value of 99%. With the exception of cluster 3, tick strains were scattered around the tree as expected. However, the bootstrap values supporting the separation of clusters 1 and 2 did not exceed 75%. Because ambiguous nucleotides were almost exclusively restricted to the most informative variable sites, a further ML tree was constructed from the 284 samples containing unambiguous bases only. It showed an increased statistical significance for the separation of the roe deer strains (Figure S3 A). The average identities and similarities between the different concatenated allele gene clusters are shown in Table 5.

**Table 5:** Net average identities^1^ and similarities^2^ between the different concatenated allele gene clusters in percent (n = 383)^3^.

	Cluster 1	Cluster 2	Cluster 3
**Cluster 1**		99,7	92,9
**Cluster 2**	*99,9*		92,9
**Cluster 3**	*96,1*	*96,2*	

1 At the nucleotide level (roman).

2 At the amino acid level (italics).

3 383 strains in which all seven loci could be amplified were included.

While we could distinguish *A. phagocytophilum* strains from the USA from those from Europe by means of MLST, they were 99% identical both at the nucleotide and amino acid level to European strains. Further, roe deer as well as vole and shrew strains were found to cluster apart from human, domestic and farm animal strains. Thus, roe deer, voles and shrews are unlikely to be reservoir hosts for granulocytic anaplasmosis in these species.

### Structure Analysis using the MLST Loci

The program STRUCTURE [38] was used to examine population clustering among the *A. phagocytophilum* strains investigated. A dataset of 518 variable sites was created consisting of all single-nucleotide polymorphisms found in the seven MLST loci. The structure analysis most strongly supported two *A. phagocytophilum* population clusters (Table 6). When the number of clusters was set to two (K = 2), STRUCTURE clustered strains from voles and shrews together, whereas all other strains comprised a second cluster (Figure S4). Evanno’s ΔK tends to be conservative [40]. Thus, smaller numbers of clusters representing the most highly differentiated sets of populations tend to be favored by this method. When the number of clusters was set to three (K = 3), *A. phagocytophilum* strains from roe deer formed a third distinct cluster, which is in agreement with the sequence-based MLST analysis. Subsequent increases in the number of clusters resulted in a further division with strains from cattle and European bison being separated from strains from humans, dogs, horses, wild boars and hedgehogs. *A. phagocytophilum* strains from sheep and red deer exhibited an admixture between these two clusters. Interestingly, when the number of clusters was set to four (K = 4), human strains from the USA clustered most closely with strains from cattle and European bison and not with the other European strains from humans. As seen in the sequence-based MLST analysis, *A. phagocytophilum* strains from ticks clustered with multiple groups.

**Table 6:** Results of the STRUCTURE analysis using the sequences of the seven MLST loci of the *A. phagocytophilum strains* (n = 383).

K^1^	μ LnP(K)^2^	s LnP(K)^3^	Ln’(K)^4^	|Ln″(K)|^5^	ΔK^6^
1	−48542.40	1.1292	na^7^	na	na
**2** 8	−**23972.18**	**9.6911**	**24570.22**	**15736.74**	**1623.838**
3	−15138.70	26.3062	8833.48	7599.12	288.872
4	−13904.34	28.4786	1234.36	1898.04	66.648
5	−14568.02	71.7684	−663.68	1045.22	14.568
6	−14186.48	35.8747	381.54	942.08	26.260
7	−14747.02	13.1885	−560.54	64.60	4.898
8	−15242.96	21.2357	−495.94	257.66	12.133
9	−15996.56	129.1626	−753.60	na	na

1 K = number of clusters assumed. Each value of K (1–9) was run five independent times.

2 μ LnP(K) = mean of the estimated log-likelihood probability of the data for K.

3 s = standard deviation of the estimated log-likelihood probability of the data for K.

4 Ln’(K) = rate of change of the mean likelihood distribution.

5 |Ln″(K)| = absolute value of the second-order change of the mean likelihood distribution.

6 ΔK = mean |Ln″ (K)| divided by the standard deviation of L(K).

7 na = not applicable.

8 The solution most strongly supported is shown in bold.

### 16S rRNA Gene-based Typing

In the past, the 16S rRNA gene was used most often for the molecular characterization of *A. phagocytophilum* [22]. Therefore, the infecting 16S rRNA gene variant was determined in all 391 samples. In 22 (6%) of these samples ambiguous nucleotides indicating double infections were found. Host species and identities of the detected 16S rRNA gene variants to GenBank entries are shown in Table S4. The phylogenetic analysis using either all strains or those containing unambiguous nucleotides only did not reveal a clear clustering of the strains according to their host species origin (Figures S2 B and S3 B). The bootstrap support of the branches was generally low. In particular, roe deer as well as vole and shrew strains were not sufficiently separated from strains of other host origin. Further, in contrast to the MLST analysis, *A. phagocytophilum* strains from the USA and Europe could not be distinguished by means of 16S rRNA gene-based typing. This might be due to the fact that in our data set the 16S rRNA gene was found to be highly conserved showing only nine (2%) polymorphic sites in a fragment of 497 bp, whereas the loci chosen for MLST contained 10% to 20% polymorphic positions (Table 2).

### *ankA* Gene-based Typing

The housekeeping genes used for MLST are prone to maintain their metabolic function and are therefore likely to be evolutionarily conserved. In contrast, the *ankA* gene is thought to be an effector protein of a type IV secretion system (T4SS) of *A. phagocytophilum* [53]. Thus, it might be a virulence factor and it has been hypothesized to be involved in host adaptation underlying diversifying selection [24], [25]. Accordingly, the *ankA* gene was found to be more diverse, showing 330 (61%) polymorphic sites (Table 2). 23 samples had to be excluded from the allele-based analysis, because the *ankA* sequences contained ambiguous nucleotides.

The phylogenetic analyses of *ankA* using either all strains or those containing unambiguous nucleotides only revealed similar results (Figures S2 C and S3 C) separating the *A. phagocytophilum* strains into five *ankA* gene clusters as has been described earlier [24], [25], [34]. Major branches were supported by bootstrap values of up to 100%. As for the MLST analysis *A. phagocytophilum* strains from the USA were found to cluster with strains from Europe, but were found on a separate branch with strong bootstrap support. Strains from the USA were 97% identical at the nucleotide level and 96% similar at the amino acid level to those from Europe. The average identities and similarities between the different *ankA* gene clusters are shown in Table 7.

**Table 7:** Net average identities^1^ and similarities^2^ between the different *ankA* gene clusters in percent (n = 392)^3^.

	Cluster 1	Cluster 2	Cluster 3	Cluster 4	Cluster 5
**Cluster 1**		81,6	60,8	54,7	60,2
**Cluster 2**	*77,4*		72,3	61,1	62,6
**Cluster 3**	*34,8*	*46,4*		50,0	54,8
**Cluster 4**	*45,3*	*43,7*	*11,9*		81,3
**Cluster 5**	*47,9*	*46,3*	*21,7*	*66,5*	

1 At the nucleotide level (roman).

2 At the amino acid level (italics).

3 392 sequences from 386 strains were included.

The distribution of the *A. phagocytophilum* strains into five *ankA* gene clusters according to their host species origin is shown in Table 8. In 6 of the 386 samples analyzed *ankA* sequences belonging to two different *ankA* gene clusters were found (2 roe deer: cluster 2 and cluster 3, 1 roe deer: cluster 2 and cluster 4, 1 red deer: cluster 3 and cluster 4, 1 cattle: cluster 1 and cluster 4, 1 tick: cluster 2 and cluster 4). With the exception of cluster 5, tick strains were as expected scattered around the tree. Strains from voles and shrews were exclusively found in cluster 5, whereas those from roe deer were part of cluster 2 (33/46), cluster 3 (9/46) and cluster 4 (4/46). On the other hand, cluster 5 was restricted to vole and shrew strains and clusters 2 and 3 with the exception of 2 red deer samples (1 of them from a sample with a double infection) to roe deer and ticks. In contrast to the MLST analysis where the *A. phagocytophilum* strains from farm and large wild animals with the exception of roe deer clustered together with strains from humans and domestic animals, a different pattern was found for the *ankA* gene-based analysis. Here, strains from sheep, cattle, red deer, European bison and chamois belonged to two different *ankA* gene clusters, cluster 1 and cluster 4. Nevertheless, the results from the MLST analysis indicating that vole and shrew strains and most of the roe deer strains are genetically distinct from those causing diseases in humans, domestic and farm animals could be confirmed.

**Table 8:** *ankA* gene cluster and host species of the *A. phagocytophilum* strains, in which *ankA* could be amplified (n = 386).

*ankA* gene cluster	Number^1^	Host species	*ankA* gene cluster	Number	Host species
NT^2^	6	3 (49) roe deer^3^	4	73	42 (54) sheep
		1 (7) cattle			11 (17) red deer
		1 (18) red deer			6 (15) European bison
		1 (33) ticks			4 (46) roe deer
1	225	63 (63) dogs^4^			2 (6) cattle
		42 (42) humans			2 (3) chamois
		30 (30) hedgehogs			1 (1) goat
		28 (28) horses			5 (32) ticks
		12 (54) sheep	2	45	33 (46) roe deer
		12 (12) wild boars			12 (32) ticks
		9 (15) European bison	5	27	24 (24) voles
		5 (17) red deer			3 (3) shrews
		4 (6) cattle	3	10	9 (46) roe deer
		2 (2) cats			1 (17) red deer
		2 (2) red foxes			
		1 (3) chamois			
		15 (32) ticks			

1 Prevalence of strains in the data set.

2 NT = nontypeable. In these samples sequences belonging to two different *ankA* gene clusters were found.

3 n (n) = nontypeable strains (all strains).

4 n (n) = strains belonging to the respective *ankA* gene cluster (typeable strains).

### Analysis for Recombination in the MLST Loci and *ankA*

Recombination in bacteria facilitates their adaptation to different environments [54], [55], [56]. However, recombination significantly affects phylogenetic analyses, because a single recombination event may introduce multiple nucleotide exchanges at once [54], [55], [56]. We used four separate analyses to test for recombination in each of the seven housekeeping genes and *ankA*. The tests applied were GENECONV [42], the pairwise homoplasy index (PHI) [43], maximum χ^2^ [44] and the neighborhood similarity score (NSS) [45].

Of the seven housekeeping genes, four (*glyA, mdh, dnaN, atpA)* showed no evidence of recombination in any of the four tests, whereas three (*pheS*, *fumC, sucA*) did produce a significant result, but only for the PHI test (Table 9). The GENECONV, maximum χ^2^ and NSS tests were not significant for any of the seven MLST loci. By contrast, all four tests revealed significant evidence for recombination in the *ankA* gene. The GENECONV test indicated that a 318 bp fragment from position 133 to 450 in the examined part of the *ankA* gene exhibited strong evidence of being recombined.

**Table 9:** Results of the four tests for recombination.

Gene	Unique^1^	Length(bp)	IS (all)^2^	IS (silent)	GENECONV	PHI (perm.)^3^	PHI (obs.)^4^	Max. χ^2*^	NSS^5^
*pheS*	65	438	77	47	0.3624	**0.008** 6	**0.007**	0.087	0.195
*glyA*	42	387	59	51	0.7709	0.342	0.325	0.699	0.242
*fumC*	40	411	49	38	0.1896	**0.002**	**0.001**	0.500	0.175
*mdh*	27	387	30	30	0.2889	0.609	0.487	0.059	0.595
*sucA*	51	429	72	42	0.4254	**0.009**	**0.007**	0.170	0.053
*dnaA*	46	405	56	49	0.8125	0.112	0.098	0.595	0.856
*atpA*	34	420	68	56	0.4155	0.803	0.740	0.316	0.115
*ankA*	92	516	289	21	**0.0090**	**<0.0001**	**<0.0001**	**0.006**	**<0.0001**

1 Number of unique sequences.

2 IS = informative sites.

3 PHI = pairwise homoplasy index, perm. = permutated.

4 obs. = observed. *Maximum χ^2^.

5 NSS = neighborhood similarity score.

6 Significant *p* values (α <0.05) are shown in bold.

### Analysis for Selection in the MLST Loci and *ankA*

As well as recombination, selection can impact phylogenetic and structure analyses. This would be especially true if divergent host adaptation has accelerated evolutionary changes in any of the genes we examined. Investigating the number of non-synonymous versus synonymous substitutions in our data set, we found the dN/dS ratio [46] to be <1 for all seven housekeeping genes (Table 2), indicating that stabilizing selection is most likely maintaining their metabolic function. The dN/dS ratio for *ankA* was also found to be <1, although the value was elevated compared to the MLST loci. In contrast to the result from the dN/dS ratio, the average similarities at the amino acid level between the different *ankA* gene clusters were considerably lower than the identities at the nucleotide level indicating that most polymorphic loci are non-synonymous (Table 7).

Because *ank*A may be involved in host adaptation [24], [25], we also looked for evidence of site-specific selection in this gene. To do so, the CODEML program in PAML 4 [47] was applied. The results comparing the nearly neutral model (M1a) to the model allowing positive selection (M2a), suggested that the *ankA* gene has evolved under adaptive evolution (Table 10). 31 sites showed support for positive selection (Table S5). Recombination has likely biased these patterns, but even when sites within the recombined region indicated by the GENCONV analysis (nucleotides 133 to 450) were excluded, 13 sites still appeared to have evolved under positive selection.

**Table 10:** Log-likelihood values and parameter estimates for the CODEML analysis of *ankA*.

Model	*p* 1	lnL^2^	Parameter Estimates
M0 (one-ratio)	1	−9411.51	ω = 0.469
M1a (nearly neutral)	2	−8214.16	p_0_ = 0.508, p_1_ = 0.492
			ω_0_ = 0.025, ω_1_ = 1.000
M2a (positive selection)	4	−7935.14^3^	p_0_ = 0.498, p_1_ = 0.291, p_2_ = 0.211
			ω_0_ = 0.032, ω_1_ = 1.000, ω_2_ = 7.039

1 *p* = number of free parameters in the ω distribution.

2 lnL = log-likelihood.

3 Significant difference between the M1a and M2a models based on the likelihood ratio test (LRT) test (α <0.05).

### Comparison of MLST, 16S rRNA and *ankA*-gene based Typing

Membership in one of the three concatenated allele gene clusters (Figures S2 A), as well as to one of the five *ankA* gene clusters (Figures S2 C) was included as a partition in the comparison of the different typing methods. As shown in Table 11, the typability was highest for the concatenated allele and the *ankA* gene clusters because ambiguous nucleotides do not disturb this kind of analysis. The typability was lowest for the ST-based MLST analysis. This is because this analysis cannot be applied if there are any ambiguous positions in any of the seven sequences. On the other hand, the discriminatory index used as an indication of the power of a typing method to distinguish between unrelated strains was highest for ST-based MLST and the *ankA* allele-based analyses. Further, as mentioned above, both of these typing methods were geographically informative separating *A. phagocytophilum* strains from the USA and from Europe.

**Table 11:** Tyability and discriminatory indices of MLST, 16S rRNA and *ankA* gene-based typing of *A. phagocytophilum* strains.

Method	Isolates	NT^1^	Typability	Typeable isolates	Number of types	Discriminatory index
						**(95% CI** 2 **)**
ST	383	99	74.2%	284	90	0.891
						(0.866–0.915)
CC^3^	383	160	58.2%	223	8	0.360
						(0.280–0.440)
MLST cluster^4^	383	0	100%	383	3	0.390
						(0.336–0.444)
16S rRNA	391	22	94.4%	369	14	0.787
						(0.763–0.811)
*ankA* allele	386	29	92.5%	357	86	0.891
						(0.863–0.920)
*ankA* cluster	386	6	98.4%	380	5	0.594
						(0.548–0.641)

1 NT = nontypeable.

2 CI = confidence interval.

3 CC = clonal complex.

4 Concatenated allele gene cluster.

To test for the concordance between the different typing methods, Wallace coefficients [50] were calculated. Country and host species origin of the strains were regarded as partitions. Because the *A. phagocytophilum* strains from ticks were scattered around the phylogenetic trees of the concatenated allele and *ankA* sequences, they were excluded from the analysis. Despite the geographical sampling bias in our data set, the concordance between the country of origin and all other partitions was low (Table 12). The continent of origin was not included in the analysis, because only 11 samples from the USA (10 human, 1 canine) were available. The concordance between ST and 16S rRNA gene-, *ankA* allele- and *ankA* cluster-based typing, respectively, was high with Wallace coefficients >0.75. The strongest association between host origin and typing method was found for the clonal complex, the concatenated allele and the *ankA* gene cluster.

**Table 12:** Wallace coefficients for the different typing methods (n = 357)^1^.

	Country	Host	ST	CC^2^	MLST cluster^3^	16S rRNA	*ankA* allele	*ankA* cluster
**Country**		0.215	0.211	0.729	0.582	0.229	0.178	0.463
**Host**	0.570		0.320	**0.886** 4	**0.998**	0.612	0.329	**0.833**
**ST**	0.513	0.359		**1.000**	**1.000**	**0.978**	**0.825**	**1.000**
**CC**	0.407	0.233	0.301		**1.000**	0.575	0.498	**1.000**
**MLST cluster**	0.262	0.167	0.152	**0.766**		0.325	0.202	0.640
**16S rRNA**	0.348	0.289	0.370	**0.843**	**0.936**		0.526	**0.813**
***ankA*** ** allele**	0.390	0.287	0.502	**1.000**	**1.000**	**0.982**		**1.000**
***ankA*** ** cluster**	0.310	0.218	0.221	**0.850**	**0.990**	0.430	0.288	

1 *A. phagocytophilum* strains from ticks were excluded.

2 CC = clonal complex.

3 Concatenated allele gene cluster.

4 Wallace coefficients >0.750 are shown in bold.

To make sure that this agreement was not due to chance alone, adjusted Wallace coefficients [51] were calculated. As shown in Table 13 similar results were obtained. Because only 223 of 383 strains were part of a clonal complex, the clonal complex was excluded as a partition. Again, the host origin showed the strongest relationship with the concatenated allele and *ankA* gene clusters. This indicates that *ankA* might be involved in host adaptation. A graphical representation of the concordance between the different partitions is shown in Figure S5.

**Table 13:** Adjusted Wallace coefficients for the different typing methods (n = 247)^1^.

	Country	Host	ST	MLST cluster^2^	16S rRNA	*ankA* allele	*ankA* cluster
**Country**		0.166	0.122	0.000	0.120	0.100	0.198
**Host**	0.391		0.223	**0.998** 3	0.519	0.244	**0.817**
**ST**	0.325	0.251		**1.000**	**0.967**	**0.773**	**1.000**
**MLST cluster**	0.000	0.041	0.037		0.103	0.066	0.326
**16S rRNA**	0.090	0.166	0.275	**0.799**		0.503	**0.775**
***ankA*** ** allele**	0.147	0.152	0.428	**1.000**	**0.982**		**1.000**
***ankA*** ** cluster**	0.059	0.103	0.111	**0.989**	0.304	0.201	

1 *A. phagocytophilum* strains from ticks were excluded. Only typeable strains with data for all partitions were included. The clonal complex as partition was excluded, because only 223 of 383 strains were part of a clonal complex.

2 Concatenated allele gene cluster.

3 Wallace coefficients >0.750 are shown in bold.

When the results of the sequenced-based phylogenetic analyses were compared, MLST and the *ankA*-based typing separated strains from voles and shrews as well as from roe deer from all other strains. In the sequence-based MLST analysis, *A. phagocytophilum* strains from humans, hedgehogs, domestic, farm and large wild animals except roe deer belonged to the same cluster. However, the *ankA*-based typing revealed that strains from sheep, cattle, red deer, European bison and chamois belonged to two different *ankA* gene clusters (cluster 1 and cluster 4), only one of them (cluster 1) containing the *A. phagocytophilum* strains from humans, domestic animals and hedgehogs.

## Discussion

MLST is a widely accepted method of bacterial typing that benefits from standardization, a universal nomenclature and free data exchange [23]. The development of an MLST typing scheme became possible after the whole genome sequence of *A. phagocytophilum* had been published [26]. Interpretation of earlier data on the molecular characterization of *A. phagocytophilum* is hindered by the fact that a variety of different target genes and a limited number of strains was investigated [22]. We here provide for the first time a standardized typing method that is easily transferable between different laboratories. We validated our approach on a large data set of almost 400 *A. phagocytophilum* strains and have made MLST profiles and isolate databases freely available (http://pubmlst.org/aphagocytophilum/). We showed that our MLST data were informative concerning host species and geographic origin, whereas the most widely used 16S rRNA gene-based typing was not. Thus, 16S rRNA gene-based typing does not allow to reliably define *A. phagocytophilum* genotypes as it had been shown before [24], [32], [34], [57], [58].

The major drawback of the MLST performed here was that multiple infections with different *A. phagocytophilum* strains seem to be more the rule than the exception. This phenomenon has been described previously in dogs [59], sheep [60], cattle [27], roe deer [61] and red deer [61]. In our data set farm and large wild animals, small mammals and ticks were especially prone to carrying multiple genetic variants. In contrast, in humans and domestic animals double infections were found only in three dog samples. We do not know whether the owners of the sheep, cattle and domestic animals investigated here used tick prevention measures, but large wild animals and small mammals have a high exposure to ticks, which increases their risk of multiple infections. It is not yet clear whether multiple infections with different *A. phagocytophilum* strains result from simultaneous transmission or from superinfection. Because ticks carrying different genetic variants of *A. phagocytophilum* have been described [34], simultaneous transmission might occur. However, detection limit of such variants in vivo is probably not identical as it has been shown experimentally in sheep that distinct *A. phagocytophilum* strains differ in their ability to cause peak bacteraemias [62], [63]. On the other hand, superinfection with different genetic variants has been demonstrated in a sheep infection model [64]. Therefore, both scenarios may occur in vivo. Accordingly, we detected identical as well as different STs and *ankA* alleles at various time points in the six hedgehog individuals that were sampled more than once (data not shown).

The housekeeping genes, as well as the 16S rRNA and the *ankA* gene analyzed here, are single copy genes [26]. It is therefore unlikely that a certain *A. phagocytophilum* strain possesses more than one allele that could lead to the ambiguous nucleotides that we observed in the chromatograms. However, for the related species *A. marginale* it was found that the vaccine strain *A. marginale* subsp. *centrale* was genetically heterogeneous at multiple loci [65]. If a similar phenomenon occurs in *A. phagocytophilum* as well, this would represent an alternative explanation, but in turn would question the concept of a strain as a genetically homogenous bacterial population. Because *A. phagocytophilum* replicates obligate intracellularly, clones cannot be separated easily. Thus, cell culture isolates grown from multiple infected hosts will not readily solve the problem of typing these strains.

Although the MLST loci represent only a minor portion of the bacterial genome, strain relatedness inferred from MLST was reflected by whole genome data [52]. To date, whole genome sequencing usually requires the isolation of the respective strain. Isolation of *A. phagocytophilum* in cell culture is possible, but laborious [66]. Therefore, the MLST analysis we propose here offers the advantage that it can be done using small amounts of DNA isolated directly from the infected host.

In our study, *A. phagocytophilum* strains from humans, dogs, horses, wild boar, and hedgehogs were found to be homologous because, with the exception of the North American strains, all belonged to clonal complex 1 and clustered together in the *ankA* gene-based phylogenetic analysis. In Europe, clustering of human, canine and equine strains has been observed previously amongst others using *groESL* [67] and *ankA* [24] sequences. *A. phagocytophilum* strains from wild boars and hedgehogs were equally part of clonal complex 1. This suggests they may serve as reservoir hosts for granulocytic anaplasmosis in humans and domestic animals. For wild boars, this has been proposed previously because of *groESL*- [68] and *ankA*- [69] based typing. European hedgehogs are infested with both, the hedgehog specialist *I. hexagonus* and the generalist *I. ricinus* [70]. Therefore, it has been suggested earlier that *I. ricinus* ticks fed on European hedgehogs could transfer *A. phagocytophilum* to humans and animals [29], [30].

The clustering of *msp2* sequences from humans, dogs and horses from North America has been observed previously [71]. Further, whole genome sequencing revealed that two human strains and one canine strain from the USA were closely related [72]. In our study, the canine *A. phagocytophilum* strain from the USA belonged to the same clonal complex 5 as the 10 North American strains from humans. This is in line with previous findings, although only one dog sample was available to us. In contrast to the 16S rRNA gene, MLST and *ankA*-based typing differentiated between *A. phagocytophilum* strains from Europe and the USA. Strains from the European clonal complex 1 and the North American clonal complex 5 did not share any alleles indicating transcontinental diversification. Nevertheless, human strains from both continents were highly identical, especially at non-synonymous sites. Further, all strains from humans, dogs and horses from Europe belonged to the same clonal complex. As canine and equine granulocytic anaplasmosis occurs frequently in Europe [13], [14], HGA is likely to be underdiagnosed in Europe.

Although three *A. phagocytophilum* strains from sheep belonged to the clonal complex 1, most of the ovine strains were assigned to unlinked STs. The phylogenetic analysis using the concatenated allele or *ankA* sequences revealed that the sheep strains were not as uniform as those from humans and domestic animals, but showed a higher diversity. This may suggest admixture in these strains as a similar result was seen in the STRUCTURE analysis. A higher degree of strain diversity was also found for *A. phagocytophilum* strains from cattle, red deer and European bison. Further, whereas strains from sheep, cattle, red deer and European bison were found in the same cluster in the sequence-based MLST analysis, they belonged to two different *ankA* gene clusters, cluster 1 and cluster 4. This heterogeneity of strains infecting ruminants could be the result of genetic exchange via recombination between *A. phagocytophilum* strains in multiply infected highly tick-exposed animals. Evidence that recombination might occur at least within the *ankA* gene is already available [25] and is further supported here. Another possibility is that the adaptation of *A. phagocytophilum* to humans and domestic animals occurred more recently than to sheep and cattle.

Using MLST and *ankA*-based typing, we found that red deer strains were closely related to those from humans and domestic animals. However, they did not belong to the clonal complex 1 that contained the human, canine and equine strains. This makes them less likely to serve as reservoir hosts for granuloytic anaplasmosis in humans and domestic animals, and questions earlier suggestions based on *groESL*- and *ankA*-based typing [24], [67]. The results we present here indicate that they might instead harbour *A. phagocytophilum* strains infectious for sheep and cattle. However, their role as reservoir hosts for granulocytic anaplasmosis in sheep has been critically debated recently using *msp4*-based typing [73].

As we found for sheep strains, roe deer strains were mostly assigned to unlinked STs. The phylogenetic analysis using the concatenated allele sequences revealed that all of them were part of the same cluster. With the exception of one *A. phagocytophilum* strain from a red deer, this cluster contained only strains from roe deer and ticks. A similar observation was made in the *ankA* gene-based phylogenetic analysis, where roe deer strains were found almost exclusively in *ankA* gene clusters 2 and 3 indicating that roe deer are unlikely to be the reservoir for granulocytic anaplasmosis in humans, domestic and farm animals as it has been suggested previously [24], [67].

In contrast to roe deer, all *A. phagocytophilum* strains from vole and shrews were part of the clonal complex 2. They were only distantly related to all other strains investigated showing a limited identity of 93% at the nucleotide level to the concatenated allele sequences of the rest of the strains. Voles and shrews are therefore unlikely to serve as reservoir hosts for granulocytic anaplasmosis in humans, domestic and farm animals. This has already been suggested before based on *groESL*- [74] and *ankA*-based typing [25] and contrasts with North America where the white-footed mouse (*Peromyscus leucopus*) is a well established reservoir species [18], [19]. Instead, in Europe voles might be involved in a separate enzootic cycle probably with *I. trianguliceps* as the tick vector [32]. Although, there might be some sampling error in our data set, we did not find any of the *A. phagocytophilum* strains from *I. ricinus* ticks analyzed here clustering with the strains from voles and shrews supporting this hypothesis.

Analyzing the population structure of *A. phagocytophilum* we found it most likely to be semiclonal with a high proportion of relatively rare unlinked STs superimposed by a limited number of rather frequent genotypes. There was one major ‘epidemic’ clonal complex containing all strains from humans, dogs and horses that might have evolved more recently than the great variety of the other unlinked STs. Our results indicate that some recombination may have occurred in three of the seven MLST loci. This recombination may disturb a fully clonal population structure in *A. phagocytophilum*. However, the support for recombination was not strong, because only one of the four tests performed was significant. Further, the phylogenetic analyses were only minimally influenced when these three genes were removed from the dataset and none of the major clusters was affected (data not shown).

Comparing the concordance between the three different typing methods used here, we observed a clear relationship between all of them. Variation in the 16S rRNA gene might therefore be linked with other loci such as certain housekeeping genes or *ankA*, but used alone, it is not sufficient for reliable typing. Whereas the MLST loci represent the core genome [52], *ankA* has been hypothesized to be involved in host adaptation underlying diversifying selection [24], [25]. This may limit its suitability for typing *A. phagocytophilum* strains, especially as recombination may facilitate host-specific adaptation and obscure phylogenetic signatures. Nevertheless, although we observed a huge diversity within this gene, the *ankA*-based typing provided similar results as the MLST analysis in separating strains from roe deer as well as from voles and shrews from all other strains. Whether these molecular data are reflected by in vivo differences should be investigated in cross-infection experiments. However, the *A. phagocytophilum* strains used should be carefully typed because the in vivo data available to date have been limited by incomplete molecular characterization of the isolates.

Although our MLST analysis was hindered by the occurrence of multiple infections, we found it a valuable new tool to investigate the genetic variation in *A. phagocytophilum*. As our analyses were not designed as an epidemiological study and because of a considerable collection bias in our data set, our results have to be confirmed by further investigations with a structured sampling plan. We hope that others might submit further profiles and isolates data to http://pubmlst.org/aphagocytophilum/in order to increase our knowledge concerning the population structure of this emerging pathogen.

## Conclusions

Based on this study, we conclude that HGA is likely to be underdiagnosed in Europe. Further, we provide evidence that wild boars and hedgehogs may serve as reservoir hosts for granulocytic anaplasmosis in humans and domestic animals in Europe. Red deer strains were found to be less uniform, but red deer may harbour *A. phagocytophilum* strains infectious for sheep and cattle. In contrast, roe deer, voles and shrews are unlikely to be reservoir hosts for granulocytic anaplasmosis in humans, domestic or farm animals. Lastly, the *ankA* gene is presumably involved in host-specific adaptation of *A. phagocytophilum* strains that probably occurred via both recombination and positive selection.

## Supporting Information

Figure S1 **Population snapshot of all detected 90 STs calculated by eBURST.**
(PPT)

Figure S2 **ML phylogenetic trees including** ***A. phagocytophilum*** **strains with ambiguous nucleotides.**
(PPT)

Figure S3 **ML phylogenetic trees including** ***A. phagocytophilum*** **strains without ambiguous nucleotides only.**
(PPT)

Figure S4 **DISTRUCT plots for runs of STRUCTURE assuming two to five clusters (K = 2–5).**
(PPT)

Figure S5 **Graphical representation of the concordance between the different partitions.**
(PPT)

Table S1 **Host species and geographic origin of the** ***A. phagocytophilum*** **positive samples (n = 391).**
(DOC)

Table S2 **Primers used for MLST of** ***A. phagocytophilum*** **.**
(DOC)

Table S3 **Nucleotide sequences of primers used for MLST of** ***A. phagocytophilum*** **.**
(DOC)

Table S4 **Identities of the detected 16S rRNA gene variants to GenBank entries and host species of the** ***A. phagocytophilum*** **positive samples (n = 391).**
(DOC)

Table S5 **Positively selected sites in the** ***ankA*** **protein based on the CODEML analysis.**
(DOC)
