# Supplementary material for: Analysis of the Population Structure of Anaplasma phagocytophilum Using Multilocus Sequence Typing
Source: PLoS One. 2014 Apr 3;9(4):e93725. doi: 10.1371/journal.pone.0093725 (PMC3974813; doi:10.1371/journal.pone.0093725)
Supplement: Figure S1 — Population snapshot of all detected 90 STs calculated by eBURST. (PPT) [file pone.0093725.s001.ppt]

## Slide 1
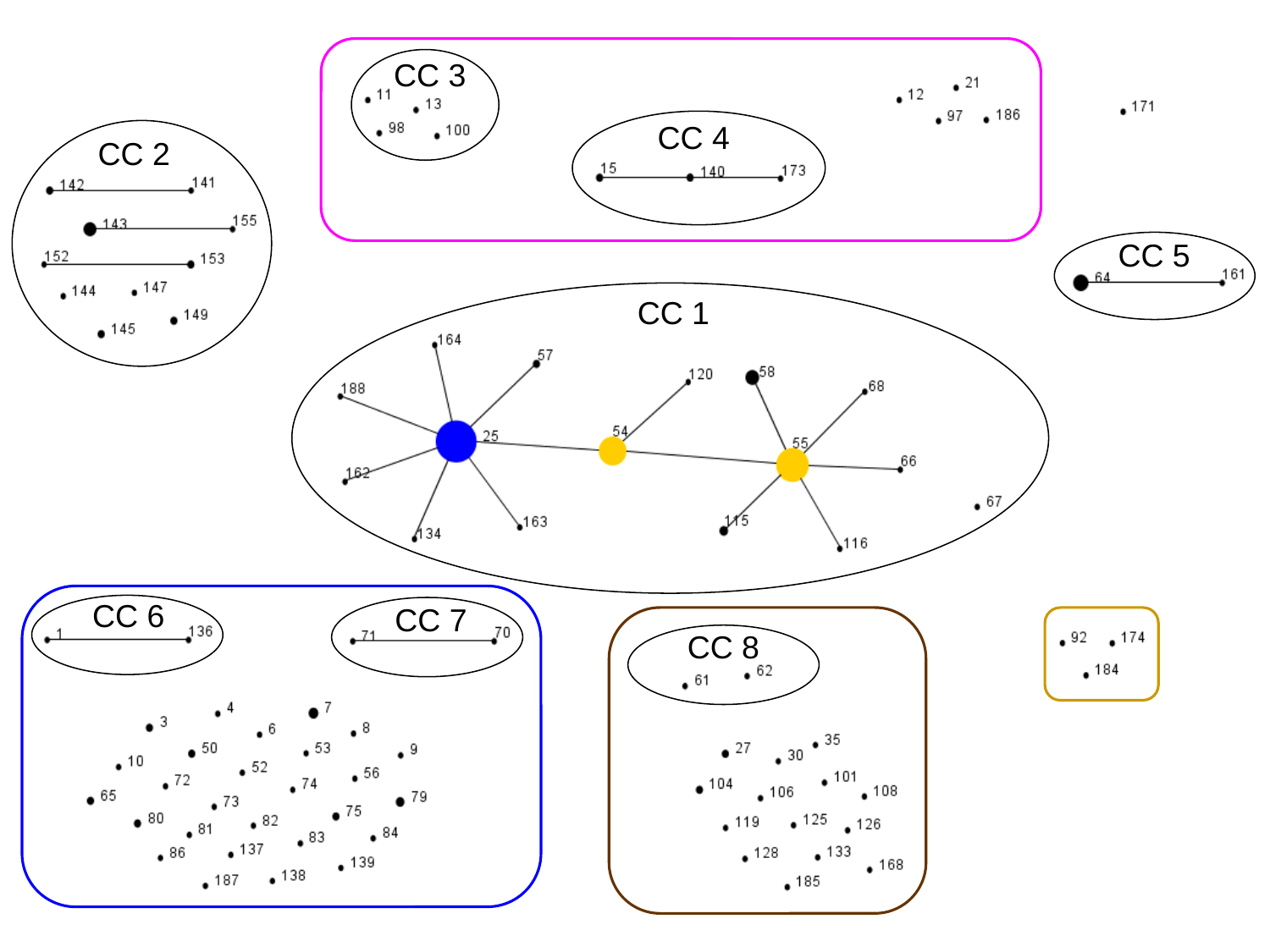

CC 3
CC 4
CC 2
CC 5
CC 1
CC 6
CC 7
CC 8

## Slide 2
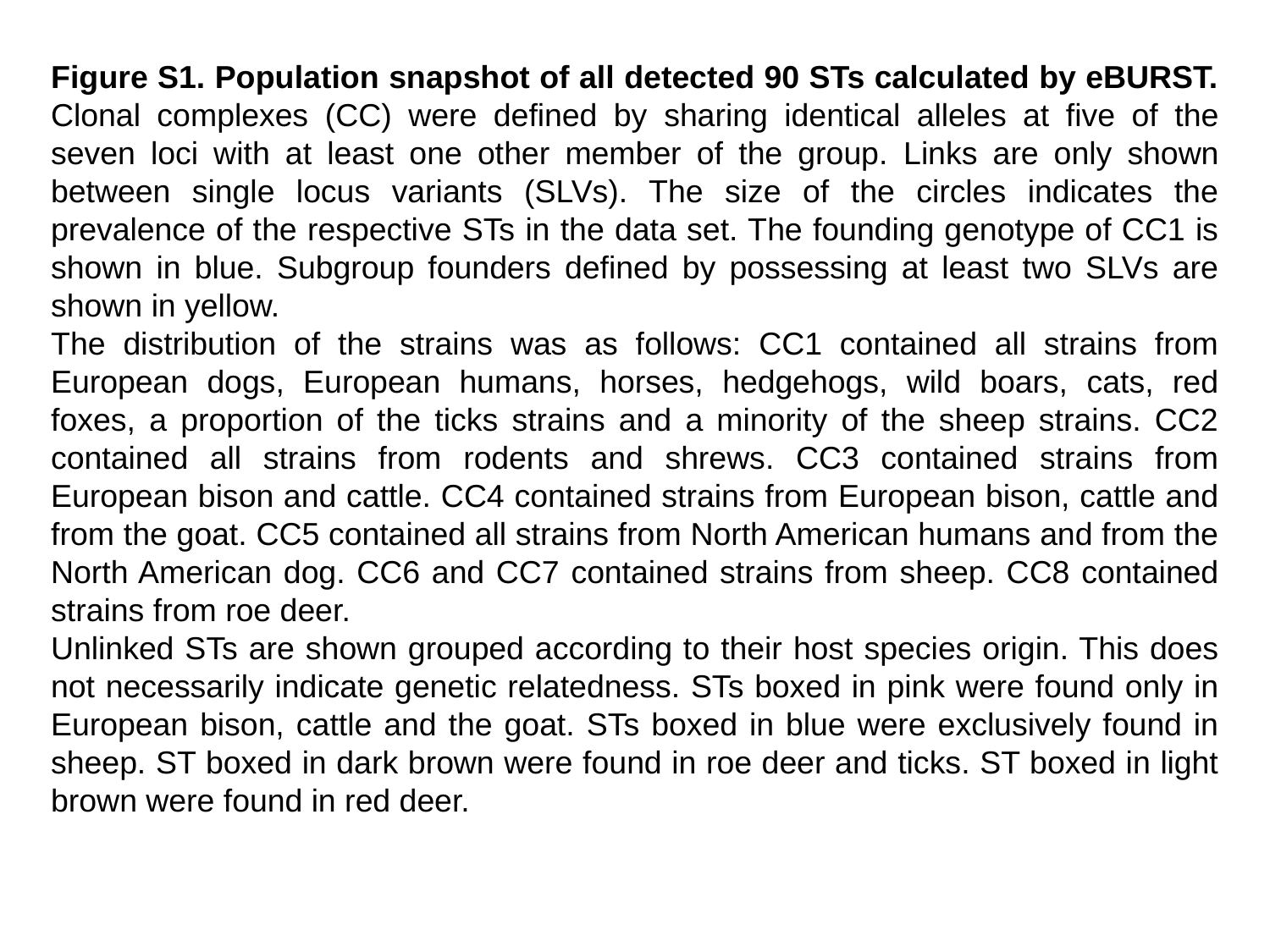

Figure S1. Population snapshot of all detected 90 STs calculated by eBURST.
Clonal complexes (CC) were defined by sharing identical alleles at five of the seven loci with at least one other member of the group. Links are only shown between single locus variants (SLVs). The size of the circles indicates the prevalence of the respective STs in the data set. The founding genotype of CC1 is shown in blue. Subgroup founders defined by possessing at least two SLVs are shown in yellow.
The distribution of the strains was as follows: CC1 contained all strains from European dogs, European humans, horses, hedgehogs, wild boars, cats, red foxes, a proportion of the ticks strains and a minority of the sheep strains. CC2 contained all strains from rodents and shrews. CC3 contained strains from European bison and cattle. CC4 contained strains from European bison, cattle and from the goat. CC5 contained all strains from North American humans and from the North American dog. CC6 and CC7 contained strains from sheep. CC8 contained strains from roe deer.
Unlinked STs are shown grouped according to their host species origin. This does not necessarily indicate genetic relatedness. STs boxed in pink were found only in European bison, cattle and the goat. STs boxed in blue were exclusively found in sheep. ST boxed in dark brown were found in roe deer and ticks. ST boxed in light brown were found in red deer.
