# Supplementary material for: Analysis of the Population Structure of Anaplasma phagocytophilum Using Multilocus Sequence Typing
Source: PLoS One. 2014 Apr 3;9(4):e93725. doi: 10.1371/journal.pone.0093725 (PMC3974813; doi:10.1371/journal.pone.0093725)
Supplement: Figure S2 — ML phylogenetic trees including A. phagocytophilum strains with ambiguous nucleotides. (PPT) [file pone.0093725.s002.ppt]

## Slide 1
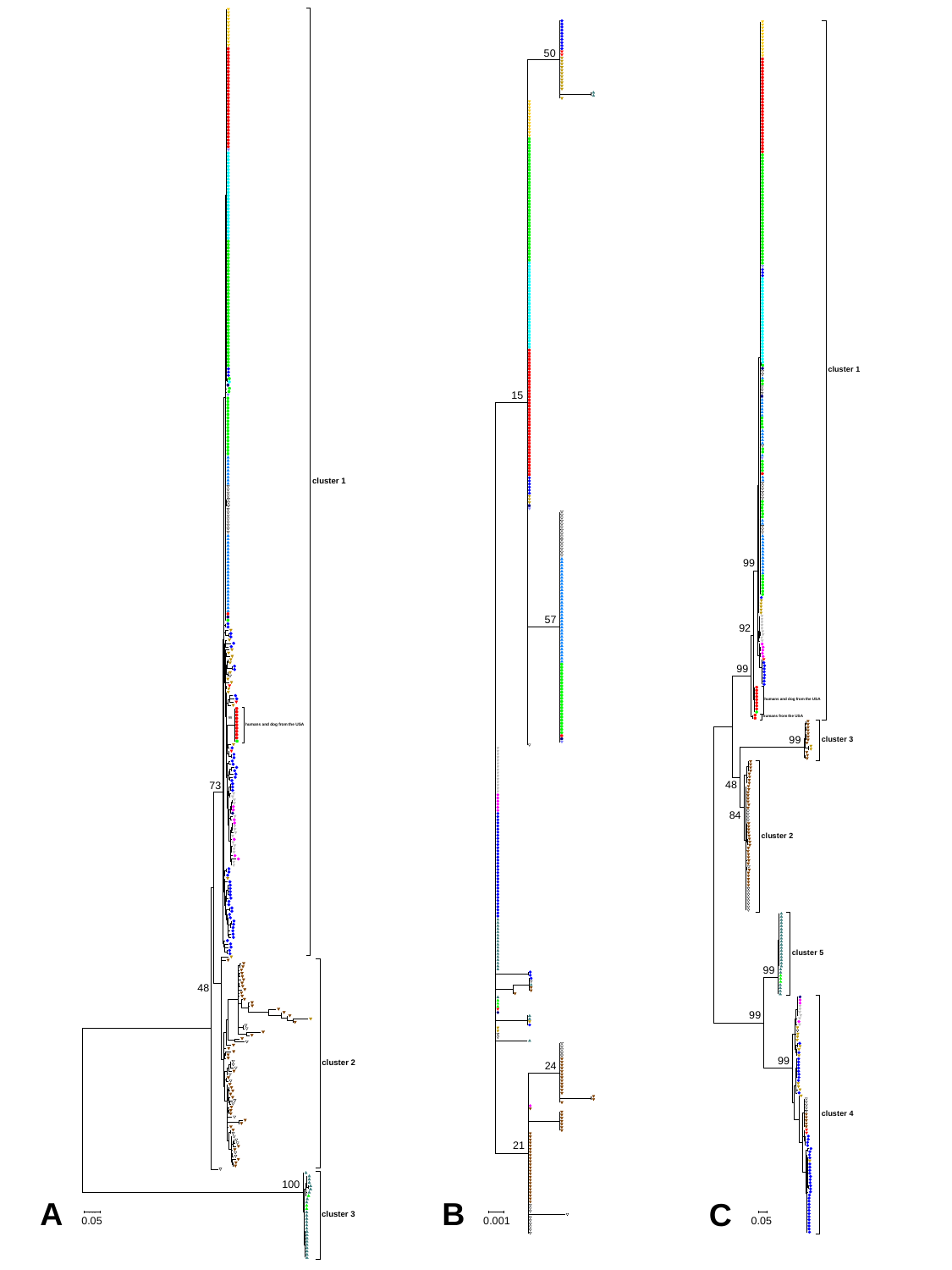

99
73
48
100
50
15
57
24
21
B
0.001
99
92
99
99
48
84
99
99
99
C
0.05
A
0.05

## Slide 2
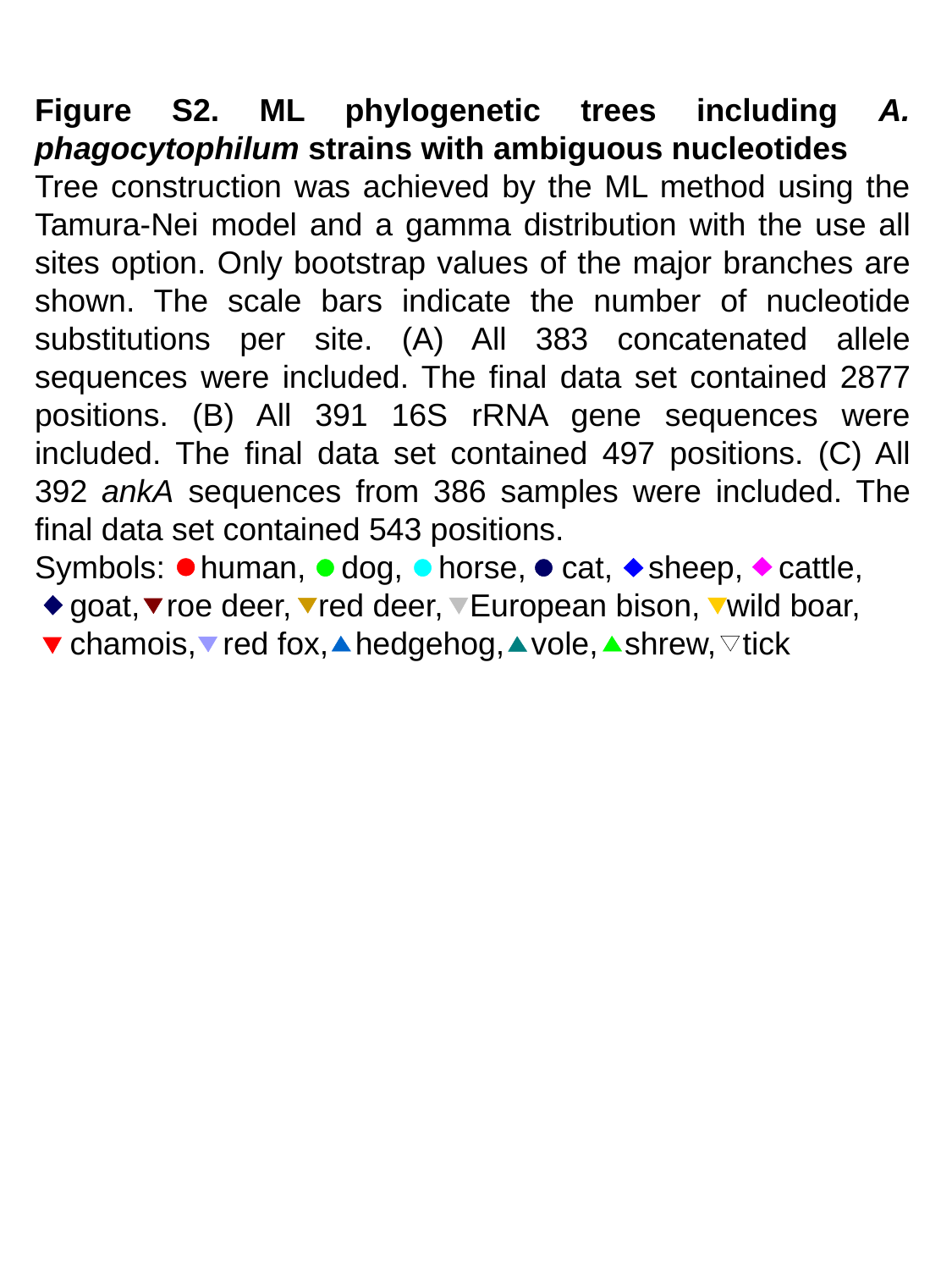

Figure S2. ML phylogenetic trees including A. phagocytophilum strains with ambiguous nucleotides
Tree construction was achieved by the ML method using the Tamura-Nei model and a gamma distribution with the use all sites option. Only bootstrap values of the major branches are shown. The scale bars indicate the number of nucleotide substitutions per site. (A) All 383 concatenated allele sequences were included. The final data set contained 2877 positions. (B) All 391 16S rRNA gene sequences were included. The final data set contained 497 positions. (C) All 392 ankA sequences from 386 samples were included. The final data set contained 543 positions.
Symbols: human, dog, horse, cat, sheep, cattle, aagoat, roe deer, red deer, European bison, wild boar, aachamois, red fox, hedgehog, vole, shrew, tick
