# Supplementary material for: Analysis of the Population Structure of Anaplasma phagocytophilum Using Multilocus Sequence Typing
Source: PLoS One. 2014 Apr 3;9(4):e93725. doi: 10.1371/journal.pone.0093725 (PMC3974813; doi:10.1371/journal.pone.0093725)
Supplement: Figure S3 — ML phylogenetic trees including A. phagocytophilum strains without ambiguous nucleotides only. (PPT) [file pone.0093725.s003.ppt]

## Slide 1
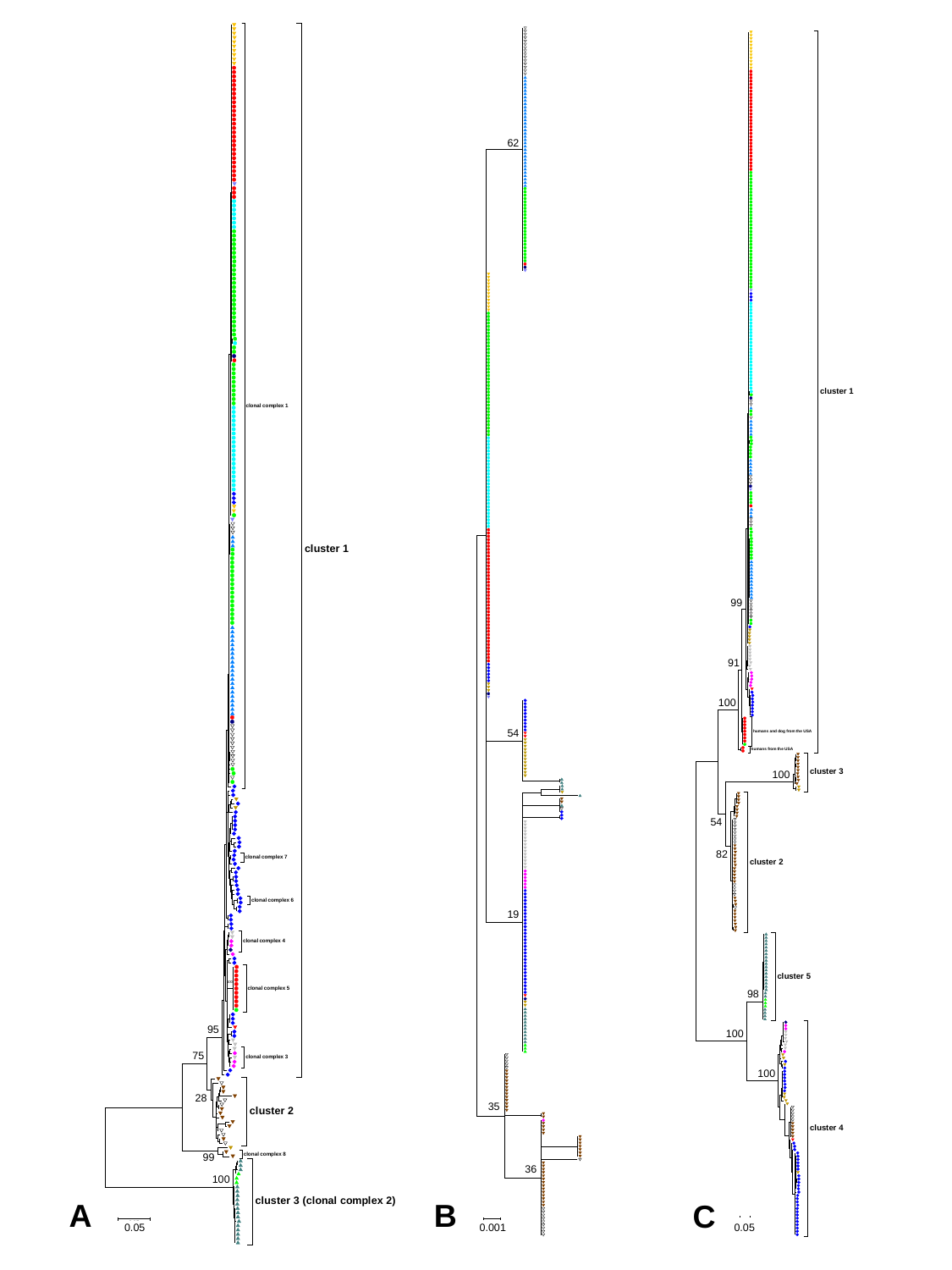

100
95
75
28
99
100
A
0.05
62
54
19
35
36
B
0.001
99
91
100
100
54
82
98
100
100
C
0.05

## Slide 2
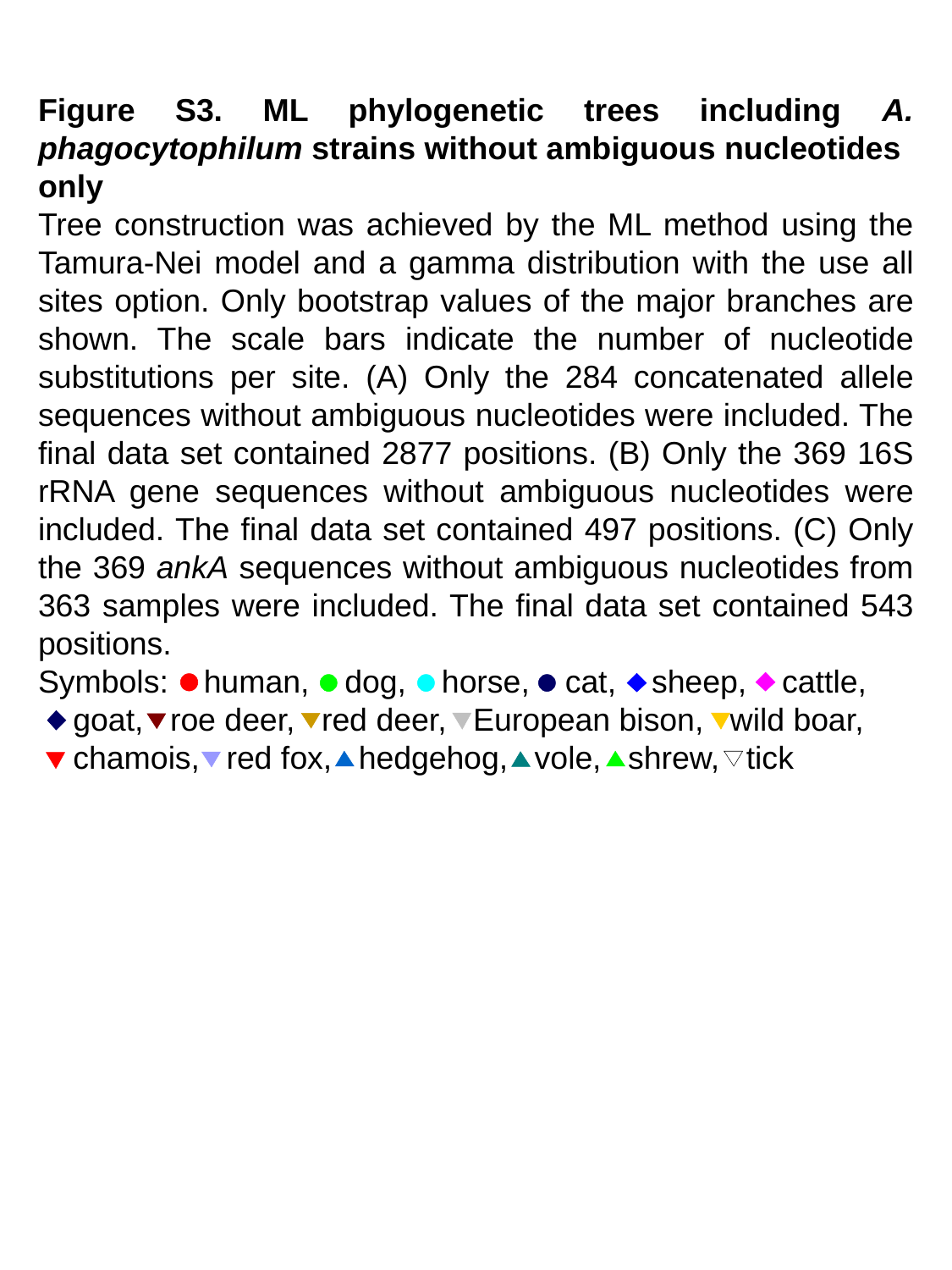

Figure S3. ML phylogenetic trees including A. phagocytophilum strains without ambiguous nucleotides
only
Tree construction was achieved by the ML method using the Tamura-Nei model and a gamma distribution with the use all sites option. Only bootstrap values of the major branches are shown. The scale bars indicate the number of nucleotide substitutions per site. (A) Only the 284 concatenated allele sequences without ambiguous nucleotides were included. The final data set contained 2877 positions. (B) Only the 369 16S rRNA gene sequences without ambiguous nucleotides were included. The final data set contained 497 positions. (C) Only the 369 ankA sequences without ambiguous nucleotides from 363 samples were included. The final data set contained 543 positions.
Symbols: human, dog, horse, cat, sheep, cattle, aagoat, roe deer, red deer, European bison, wild boar, aachamois, red fox, hedgehog, vole, shrew, tick
