# Supplementary material for: Analysis of the Population Structure of Anaplasma phagocytophilum Using Multilocus Sequence Typing
Source: PLoS One. 2014 Apr 3;9(4):e93725. doi: 10.1371/journal.pone.0093725 (PMC3974813; doi:10.1371/journal.pone.0093725)
Supplement: Figure S4 — DISTRUCT plots for runs of STRUCTURE assuming two to five clusters (K = 2–5). (PPT) [file pone.0093725.s004.ppt]

## Slide 1
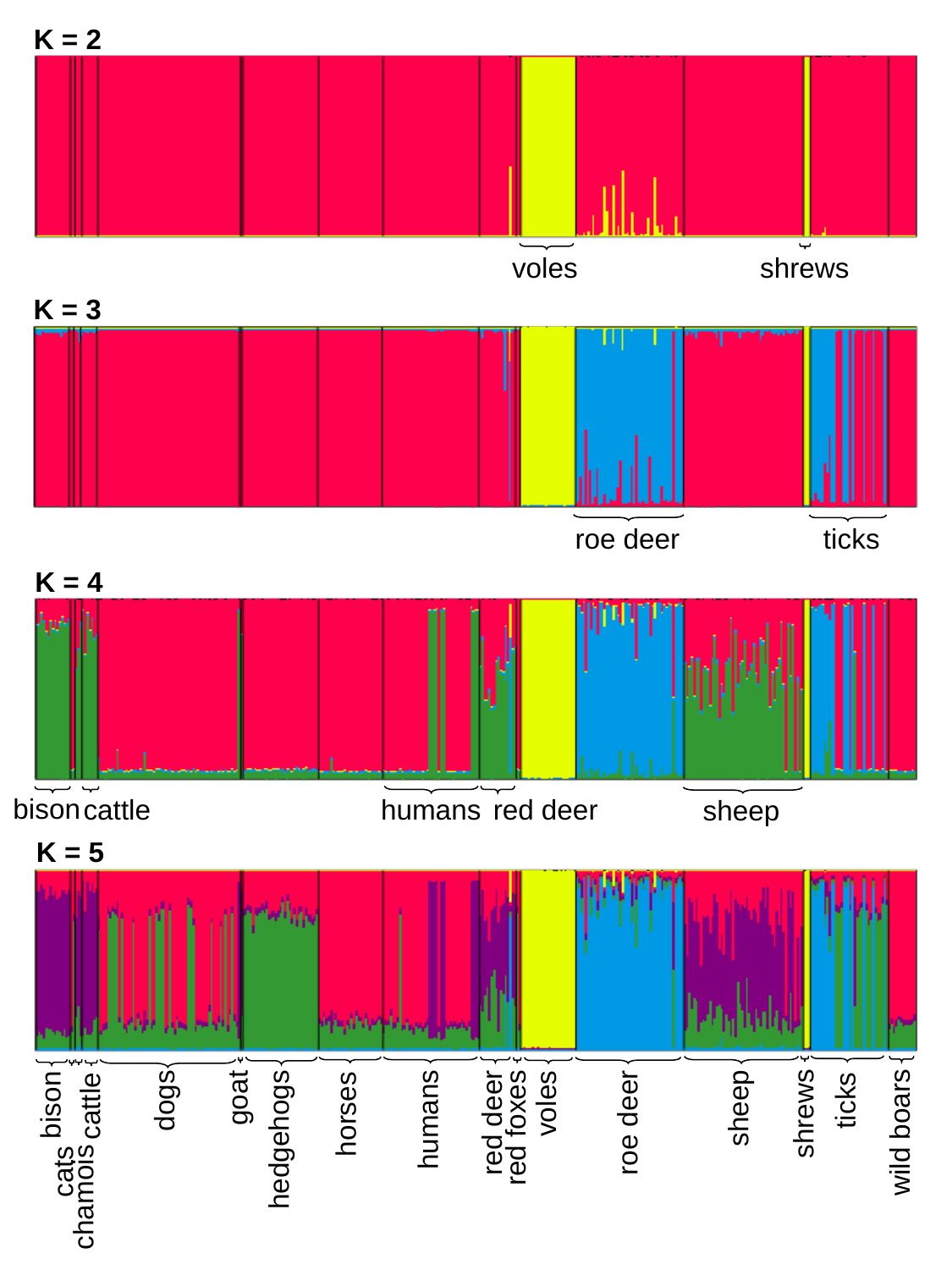

K = 2
voles
shrews
K = 3
roe deer
ticks
K = 4
bison
red deer
cattle
humans
sheep
K = 5
goat
ticks
dogs
voles
bison
cattle
sheep
shrews
horses
humans
red deer
roe deer
red foxes
wild boars
hedgehogs
cats
chamois

## Slide 2
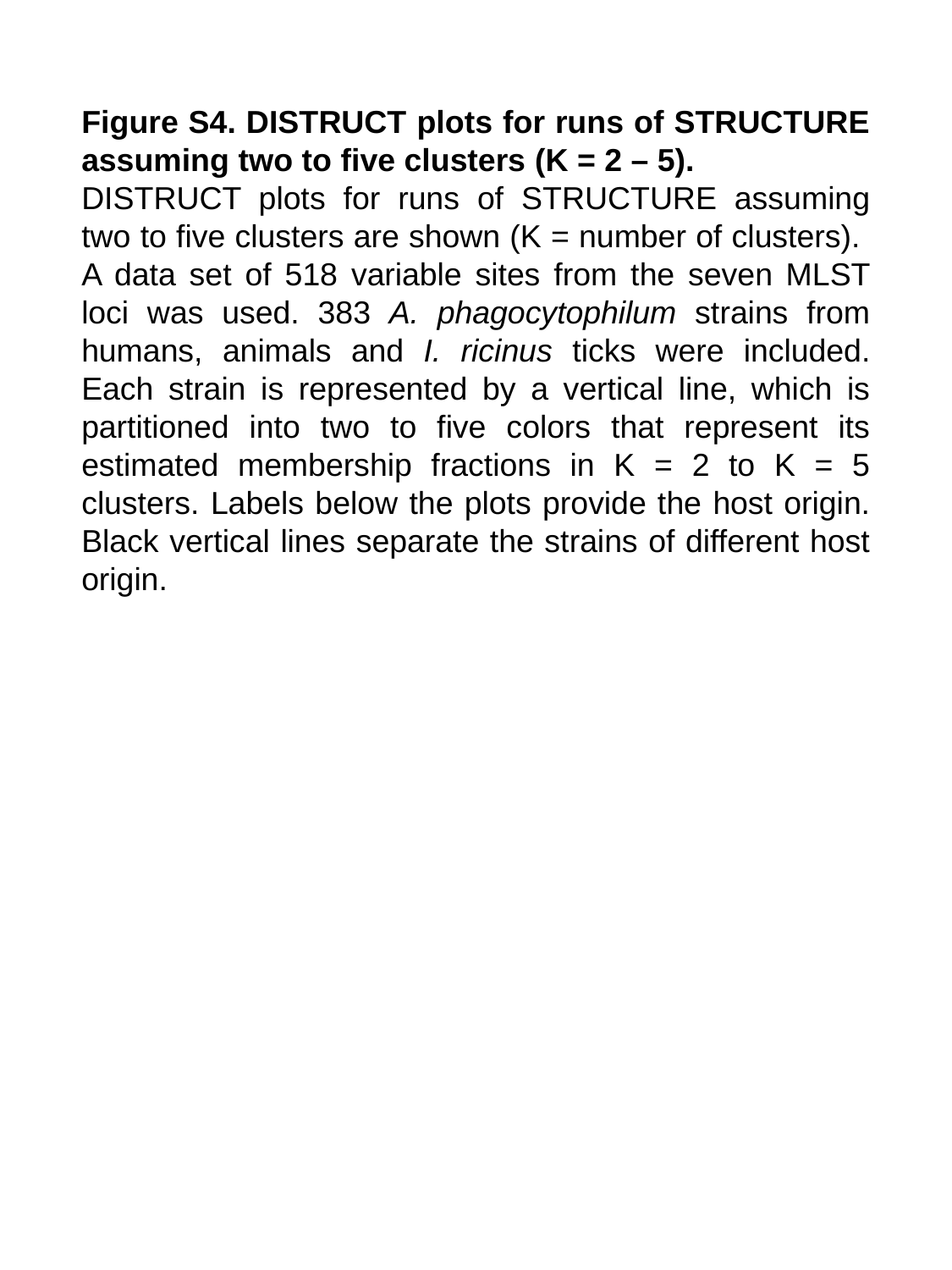

Figure S4. DISTRUCT plots for runs of STRUCTURE assuming two to five clusters (K = 2 – 5).
DISTRUCT plots for runs of STRUCTURE assuming two to five clusters are shown (K = number of clusters). A data set of 518 variable sites from the seven MLST loci was used. 383 A. phagocytophilum strains from humans, animals and I. ricinus ticks were included. Each strain is represented by a vertical line, which is partitioned into two to five colors that represent its estimated membership fractions in K = 2 to K = 5 clusters. Labels below the plots provide the host origin. Black vertical lines separate the strains of different host origin.
