# Supplementary material for: Analysis of the Population Structure of Anaplasma phagocytophilum Using Multilocus Sequence Typing
Source: PLoS One. 2014 Apr 3;9(4):e93725. doi: 10.1371/journal.pone.0093725 (PMC3974813; doi:10.1371/journal.pone.0093725)
Supplement: Figure S5 — Graphical representation of the concordance between the different partitions. (PPT) [file pone.0093725.s005.ppt]

## Slide 1
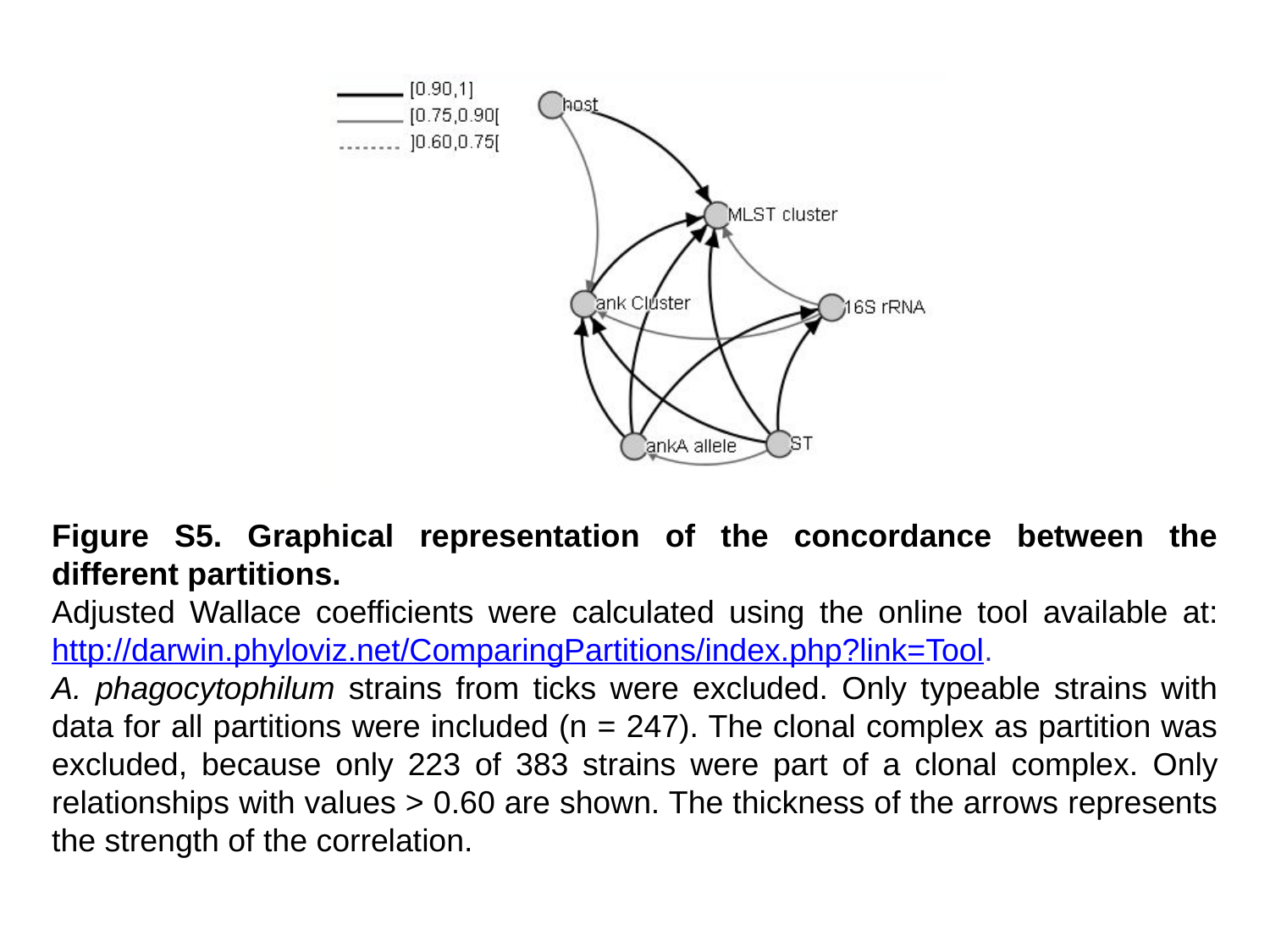

Figure S5. Graphical representation of the concordance between the different partitions.
Adjusted Wallace coefficients were calculated using the online tool available at: http://darwin.phyloviz.net/ComparingPartitions/index.php?link=Tool.
A. phagocytophilum strains from ticks were excluded. Only typeable strains with data for all partitions were included (n = 247). The clonal complex as partition was excluded, because only 223 of 383 strains were part of a clonal complex. Only relationships with values > 0.60 are shown. The thickness of the arrows represents the strength of the correlation.
