# Supplementary material for: Analysis of the Population Structure of Anaplasma phagocytophilum Using Multilocus Sequence Typing
Source: PLoS One. 2014 Apr 3;9(4):e93725. doi: 10.1371/journal.pone.0093725 (PMC3974813; doi:10.1371/journal.pone.0093725)
Supplement: Table S1 — Host species and geographic origin of the A. phagocytophilum positive samples (n = 391). (DOC) [file pone.0093725.s006.doc]

**Table S1.** Host species and geographic origin of the *A. phagocytophilum* positive samples (n = 391)

| **Group (n)** | **Host species (n)** | **Geographic origin (n)** | **Reference** |
| --- | --- | --- | --- |
| Humans (42) | *Homo sapiens* (42) | Slovenia (32) | [1], this study |
|  |  | USA (10) | [1,2,3] |
| Domestic animals (93) | *Canis lupus familiaris* (63) | Germany (37) | [1], this study |
|  |  | Slovenia (10) | [1], this study |
|  |  | Switzerland (8) | [1], this study |
|  |  | Austria (2) | [1] |
|  |  | Sweden (2) | [1] |
|  |  | Denmark (1) | [1] |
|  |  | France (1) | This study |
|  |  | Spain (1) | This study |
|  |  | USA (1) | [2] |
|  | *Equus caballus* (28) | Germany (22) | [1], this study |
|  |  | Switzerland (3) | [1,2], this study |
|  |  | Denmark (2) | This study |
|  |  | The Netherlands (1) | This study |
|  | *Felis catus* (2) | Austria (1) | [1] |
|  |  | Switzerland (1) | [1] |
| Farm animals (62) | *Ovis aries* (54) | Norway (42) | [1] |
|  |  | Germany (12) | [1,2] |
|  | *Bos taurus* (7) | Germany (3) | [2,4] |
|  |  | Norway (2) | [1] |
|  |  | Spain (2) | [1] |
|  | *Capra aegagrus hircus* (1) | Germany (1) | [2] |
| Large wild animals (99) | *Capreolus capreolus* (49) | Germany (29) | [1] |
|  |  | Slovenia (14) | [1], this study |
|  |  | Spain (4) | [1] |
|  |  | Norway (2) | [1] |
|  | *Cervus elaphus* (18) | Slovenia (11) | [1], this study |
|  |  | Germany (3) | [1], this study |
|  |  | Poland (3) | [1] |
|  |  | Norway (1) | [1] |
|  | *Bison bonasus* (15) | Poland (15) | [1] |
|  | *Sus scrofa* (12) | Slovenia (12) | This study |
|  | *Rupicapra rupicapra* (3) | Slovenia (3) | This study |
|  | *Vulpes vulpes* (2) | Germany (2) | [5] |
| Small mammals (61) | *Erinaceus europaeus* (34) | Germany (34) | [6,7], this study |
|  | *Myodes glareolus* (19) | Germany (19) | [2] |
|  | *Mircotus arvalis* (3) | Germany (3) | [2] |
|  | *Microtus agrestis* (2) | UK (2) | [2] |
|  | *Sorex araneus* (3) | UK (3) | [2] |
| Ticks (34) | *I. ricinus* (34) | Germany (34) | [1], this study |

1. Scharf W, Schauer S, Freyburger F, Petrovec M, Schaarschmidt-Kiener D, et al. (2011) Distinct host species correlate with *Anaplasma phagocytophilum ankA* gene clusters. J Clin Microbiol 49: 790-796.

2. Majazki J, Wüppenhorst N, Hartelt K, Birtles R, von Loewenich FD (2013) *Anaplasma phagocytophilum* strains from small mammals exhibit specific *ankA* gene sequences. BMC Vet Res 9: 235.

3. Dunning Hotopp JC, Lin M, Madupu R, Crabtree J, Angiuoli SV, et al. (2006) Comparative genomics of emerging human ehrlichiosis agents. PLoS Genet 2: e21.

4. Henniger T, Henniger P, Grossmann T, Distl O, Ganter M, et al. (2013) Congenital infection with *Anaplasma phagocytophilum* in a calf in northern Germany. Acta Vet Scand 55: 38.

5. Härtwig V, von Loewenich FD, Schulze C, Straubinger RK, Daugschies A, et al. (2014) Detection of *Anaplasma phagocytophilum* in red foxes (*Vulpes vulpes*) and racoon dogs (*Nyctereutes procyonoides*) from Brandenburg, Germany. Ticks Tick Borne Dis: Epub ahead of print.

6. Skuballa J, Petney T, Pfäffle M, Taraschewski H (2010) Molecular detection of *Anaplasma phagocytophilum* in the European hedgehog (*Erinaceus europaeus*) and its ticks. Vector Borne Zoonotic Dis 10: 1055-1057.

7. Silaghi C, Skuballa J, Thiel C, Pfister K, Petney T, et al. (2012) The European hedgehog (*Erinaceus europaeus*) - a suitable reservoir for variants of *Anaplasma phagocytophlium*? Ticks Tick Borne Dis 3: 49-54.
