# Supplementary material for: Analysis of the Population Structure of Anaplasma phagocytophilum Using Multilocus Sequence Typing
Source: PLoS One. 2014 Apr 3;9(4):e93725. doi: 10.1371/journal.pone.0093725 (PMC3974813; doi:10.1371/journal.pone.0093725)
Supplement: Table S2 — Primers used for MLST of A. phagocytophilum . (DOC) [file pone.0093725.s007.doc]

**Table S2.** Primers used for MLST of *A. phagocytophilum*

| **Gene** | **First amplification** | **Nested amplification** | **Sequencing** |
| --- | --- | --- | --- |
| *pheS* | pheS 1f + pheS 2r | pheS 3f + pheS 4r | pheS 3f, pheS 4r |
|  | pheS 1f a + pheS 2r a | pheS 3f a + pheS 4r a | pheS 3f a, pheS 4r a |
|  | pheS 1f b + pheS 4r | pheS 3f b+ pheS 4r | pheS 3f b, pheS 4r |
| *glyA* | glyA 1f + glyA 2r | glyA 3f + glyA 4r | glyA 3f, glyA 4r |
|  | glyA 1f a + glyA 2r a | glyA 3f a + glyA 4r a | glyA 3f, glyA 4r a |
|  | glyA 1f a + glyA 2r a | glyA 3f b + glyA 4r a | glyA 3f b, glyA 4r a |
| *fumC* | fumC 1f + fumC 2r | fumC 3f + fumC 4r | fumC 3f, fumC 4r |
|  | fumC 1f a + fumC 2r a | fumC 3f a + fumC 4r a | fumC 3f, fumC 4r a |
|  | fumC 1f b + fumC 2r b | fumC 3f b + fumC 4r b | fumC 3f, fumC 4r b |
| *mdh* | mdh 1f + mdh 2r | mdh 3f + mdh 4r | mdh 3f, mdh 4r |
|  | mdh 1f a + mdh 2r a | mdh 3f + mdh 4r a | mdh 3f, mdh 4r a |
| *sucA* | sucA 1f + sucA 2r | sucA 3f + sucA 4r | sucA 3f, sucA 4r |
|  | sucA 1f a + sucA 2r a | sucA 3f a + sucA 4r a | sucA 3f a, sucA 4r a |
| *dnaN* | dnaN 1f + dnaN 2r | dnaN 3f + dnaN 4r | dnaN 3f, dnaN 4r |
|  | dnaN 1f + dnaN 2r | dnaN 3f a + dnaN 4r | dnaN 3f a, dnaN 4r |
|  | dnaN 1f a + dnaN 2r a | dnaN 3f b + dnaN 4r a | dnaN 3f b, dnaN 4r a |
| *atpA* | atpA 1f + atpA 2r | atpA 3f + atpA 4r | atpA 3f, atpA 4r |
|  | atpA 1f a + atpA 2r a | atpA 3f a + atpA 4r a | atpA 3f a, atpA 4r a |
