# Supplementary material for: Analysis of the Population Structure of Anaplasma phagocytophilum Using Multilocus Sequence Typing
Source: PLoS One. 2014 Apr 3;9(4):e93725. doi: 10.1371/journal.pone.0093725 (PMC3974813; doi:10.1371/journal.pone.0093725)
Supplement: Table S3 — Nucleotide sequences of primers used for MLST of A. phagocytophilum . (DOC) [file pone.0093725.s008.doc]

**Table S3.** Nucleotide sequences of primers used for MLST of *A. phagocytophilum*

| **Gene** | **Name** | **Sequence (5’ – 3’)** |
| --- | --- | --- |
| *pheS* | pheS 1f | TCT AGG CCG CGT GTA TAT G |
|  | pheS 1f a | GCA TCA GAG AGA GGC AGA AT |
|  | pheS 1f b | ATA GAA GAT ATT TTT ATT GGG CT |
|  | pheS 2r | TAA CCA GTC AAT GCG ACT AC |
|  | pheS 2r a | AAG CCG TAA TGC CCT AAC CA |
|  | pheS 3f | CAC ACC TGT ACA TCA TCC AGC C |
|  | pheS 3f a | GTA ACA AAG GTA GTC AAG GAT |
|  | pheS 3f b | GGT TTG CTA CTG TTC GTG G |
|  | pheS 4r | CAC CCA TTC CGA AAG CAA AAC C |
|  | pheS 4r a | CTG AGG TCT CCA ATG TCG T |
| *glyA* | glyA 1f | GTG ATA ATG ACA GAT GAT GAG |
|  | glyA 1f a | CGC TAA TGT TCA GCC GCA CT |
|  | glyA 2r | AAG TAT CGT ACG GCA CTA CC |
|  | glyA 2r a | CTA GCA AGC CAA AGT ATC GT |
|  | glyA 3f | TGT AAT AGC GGC GAA AGC GG |
|  | glyA 3f a | CTA GGG ATG TCA TTG GAC TC |
|  | glyA 3f b | TGT AAT AGC AGC AAA AGC AG |
|  | glyA 4r | AGC AAC CTC TTC TCT AAC CCC C |
|  | glyA 4r a | CGT ACG GCA CTA CCC TAA CA |
| *fumC* | fumC 1f | CTT ACA AGA TGC TAC TCC GT |
|  | fumC 1f a | ACT GGC TCC GGA ACG CAG |
|  | fumC 1f b | GCA GCA TTG ATG AGT CTG TG |
|  | fumC 2r | GAT TCA GCG CAG TCA CCA A |
|  | fumC 2r a | ACA ATT CTG TCG AAC TCT TCA G |
|  | fumC 2r b | GTG TGA CCA TTT TGA CAG GAT |
|  | fumC 3f | TGT CCG TTT GTG ACA GCA GAG |
|  | fumC 3f a | GTG GTG AGA TAG GAA GTA AGT |
|  | fumC 3f b | AGA GGT TGT AGA GGG CAG AT |
|  | fumC 4r | TCA GCG ATG CAC CTA GCA AAG |
|  | fumC 4r a | GAC AAG CTT CAG GGT AAC TG |
|  | fumC 4r b | TGC TGC ATT ATC ATA ACC GAT |
| *mdh* | mdh 1f | GTA TTC GTA GAT GTG ATG GA |
|  | mdh 1f a | GTG TTG CGG GTA TCT GTC A |
|  | mdh 2r | CCA CAG CTT ATA AGG TCT GA |
|  | mdh 2r a | TCC TCC CTT GCG AGT CCT |
|  | mdh 3f | TGT GCC ACG GGG GAA GTT ATT G |
|  | mdh 4r | AGG CAA CAT AAG GTC ACC GTG C |
|  | mdh 4r a | CAC ATC CTC AGA ACT CAG AC |
| *sucA* | sucA 1f | GCT TGC TAT GGA GTA TCG TG |
|  | sucA 1f a | GAA GCT GTG GTA GCA GTT AC |
|  | sucA 2r | CAA ATA TTC CGC ACC TGT AG |
|  | sucA 2r a | TTC CCA TAT CAC AAG AAC ATC |
|  | sucA 3f | ATG AGC CTA TGT TTA CGC AGC |
|  | sucA 3f a | AGA CGT TGT GAT AGA TGT GG |
|  | sucA 4r | TCT TCA CCA GAT AGA CGC ACC C |
|  | sucA 4r a | CAT GGC GGT GCG AGA AAG T |
| *dnaN* | dnaN 1f | AGA CAT ATC AAT AGA AGC GTG |
|  | dnaN 1f a | GAA GCT AAG GGC TCA GTT AT |
|  | dnaN 2r | CAA TTG ATA CAC TAT TAC CGC A |
|  | dnaN 2r a | CGC TGA ACT TAA TAC CAA CTT A |
|  | dnaN 3f | TAG TGG CGT GTG GAA ATG CGA G |
|  | dnaN 3f a | TAG TGG CGT GTG GAA ATG CGC G |
|  | dnaN 3f b | AGC GGA CGT TAG GTT CAA AC |
|  | dnaN 4r | GTC TGG AAA CGT ACC GTC CAA C |
|  | dnaN 4r a | ACT ACT GAT ACA CGG TCT AC |
| *atpA* | atpA 1f | CTC AAG ATT CTT AGA GAG AGA |
|  | atpA 1f a | ATA GAG AAC TTT GGT GGT CC |
|  | atpA 2r | ATA GCA CAA GCA GCA TAA GG |
|  | atpA 2r a | AGA AAG ATC ATC ATA TAC TAT CA |
|  | atpA 3f | ATG GAG CGG GAT TTG GAG AGA C |
|  | atpA 3f a | TAG GGG AGG TTC TTT CTG TA |
|  | atpA 4r | TTA TCC TTC TCA CTT GCC GCA C |
|  | atpA 4r a | AGT CTG ACG CAC CAG TAG C |
