# Supplementary material for: Analysis of the Population Structure of Anaplasma phagocytophilum Using Multilocus Sequence Typing
Source: PLoS One. 2014 Apr 3;9(4):e93725. doi: 10.1371/journal.pone.0093725 (PMC3974813; doi:10.1371/journal.pone.0093725)
Supplement: Table S4 — Identities of the detected 16S rRNA gene variants to GenBank entries and host species of the A. phagocytophilum positive samples (n = 391). (DOC) [file pone.0093725.s009.doc]

**Table S4.** Identities of the detected 16S rRNA gene variants to GenBank entries and host species of the *A. phagocytophilum* positive samples (n = 391)

| **16S rRNA gene variant** | **Number**^1^ | **Host species** | **16S rRNA gene variant** | **Number** | **Host species** |
| --- | --- | --- | --- | --- | --- |
| NT^2^ | 22 | 7 (49) roe deer^3^ | AF136713 | 27 | 17 (42) roe deer |
|  |  | 7 (24) rodents |  |  | 1 (7) cattle |
|  |  | 3 (54) sheep |  |  | 9 (31) ticks |
|  |  | 2 (63) dogs | AF481852 | 24 | 12 (18) red deer |
|  |  | 3 (34) ticks |  |  | 10 (51) sheep |
| U02521 | 130 | 41 (42) humans^4^ |  |  | 2 (3) chamois |
|  |  | 38 (61) dogs | AF136714 | 18 | 13 (42) roe deer |
|  |  | 28 (28) horses |  |  | 5 (31) ticks |
|  |  | 12 (12) wild boars | AF384214 | 7 | 7 (42) roe deer |
|  |  | 6 (51) sheep | AY281785 | 5 | 2 (42) roe deer |
|  |  | 3 (18) red deer |  |  | 1 (51) sheep |
|  |  | 1 (2) cats |  |  | 1 (17) rodents |
|  |  | 1 (2) red foxes |  |  | 1 (31) ticks |
| AF136712 | 75 | 34 (34) hedgehogs | GU236538 | 3 | 3 (42) roe deer |
|  |  | 23 (61) dogs | AF172166 | 3 | 2 (17) rodents |
|  |  | 1 (42) humans |  |  | 1 (18) red deer |
|  |  | 1 (2) cats | GU236611 | 2 | 2 (51) sheep |
|  |  | 1 (2) red foxes | AY082656 | 2 | 2 (17) rodents |
|  |  | 15 (31) ticks | KC740432 | 1 | 1 (17) rodents |
| M73220 | 71 | 32 (51) sheep | AY281804 | 1 | 1 (31) ticks |
|  |  | 15 (15) European bison |  |  |  |
|  |  | 11 (17) rodents |  |  |  |
|  |  | 6 (7) cattle |  |  |  |
|  |  | 3 (3) shrews |  |  |  |
|  |  | 2 (18) red deer |  |  |  |
|  |  | 1 (1) goat |  |  |  |
|  |  | 1 (3) chamois |  |  |  |

^1^Prevalence of the respective 16S rRNA gene variant in the dataset. ^2^NT = nontypeable. ^3^n (n) = nontypeable strains (all strains). ^4^n (n) = strains with respective 16S rRNA gene variant (typeable strains).
