# Supplementary material for: Analysis of the Population Structure of Anaplasma phagocytophilum Using Multilocus Sequence Typing
Source: PLoS One. 2014 Apr 3;9(4):e93725. doi: 10.1371/journal.pone.0093725 (PMC3974813; doi:10.1371/journal.pone.0093725)
Supplement: Table S5 — Positively selected sites in the ankA protein based on the CODEML analysis. (DOC) [file pone.0093725.s010.doc]

**Table S5.** Positively selected sites in the *ankA* protein based on the CODEML analysis.

| Amino acid site | Pr(w >1 )^1^ | µ^2^ | s^3^ |
| --- | --- | --- | --- |
| **4**^4^ | 0.953 | 6.748 | 1.379 |
| **9** | 1.000 | 7.046 | 0.534 |
| **12** | 1.000 | 7.046 | 0.534 |
| **25** | 1.000 | 7.046 | 0.534 |
| **26** | 1.000 | 7.046 | 0.534 |
| **29** | 1.000 | 7.046 | 0.534 |
| **31** | 1.000 | 7.046 | 0.534 |
| **32** | 1.000 | 7.046 | 0.534 |
| **33** | 1.000 | 7.046 | 0.535 |
| **35** | 1.000 | 7.046 | 0.534 |
| **40** | 0.993 | 7.001 | 0.743 |
| 57 | 1.000 | 7.046 | 0.534 |
| 58 | 0.992 | 6.996 | 0.759 |
| 60 | 1.000 | 7.046 | 0.534 |
| 61 | 1.000 | 7.046 | 0.534 |
| 65 | 0.998 | 7.037 | 0.584 |
| 72 | 0.988 | 6.975 | 0.839 |
| 85 | 0.997 | 7.025 | 0.638 |
| 86 | 1.000 | 7.046 | 0.534 |
| 87 | 0.991 | 6.993 | 0.776 |
| 95 | 1.000 | 7.046 | 0.534 |
| 110 | 1.000 | 7.046 | 0.536 |
| 116 | 1.000 | 7.046 | 0.534 |
| 120 | 0.981 | 6.928 | 0.972 |
| 121 | 1.000 | 7.045 | 0.539 |
| 125 | 1.000 | 7.046 | 0.535 |
| 129 | 1.000 | 7.046 | 0.534 |
| 140 | 1.000 | 7.046 | 0.534 |
| 149 | 1.000 | 7.046 | 0.534 |
| **160** | 1.000 | 7.046 | 0.534 |
| **171** | 1.000 | 7.046 | 0.534 |

^1^Pr(w > 1) = posterior probability. ^2^µ = mean. ^3^s= standard deviation. ^4^Positively selected sites outside the recombined region detected by GENECONV (nucleotide position 133 to 450) are shown in bold.
